# *JrMYB44* is required for the accumulation of polyphenols and contributes to drought tolerance in *Juglans regia*

**DOI:** 10.1007/s44154-024-00193-7

**Published:** 2025-01-21

**Authors:** Xiangqian Gao, Tianyu Wang, Dapei Li, Sisi Chen, Shen Yang, Chenhao Li, Siyu Hou, Muhong Xie, Zhenggang Xu, Guiyan Yang

**Affiliations:** 1 Laboratory of Walnut Research Center, College of Forestry, Northwest A and F University, Yangling, 712100 Shaanxi China; 2 Research Institute of Forestry, Chinese Academy of Forestry, Beijing, 100091 China

**Keywords:** Walnut, MYB TF, Polyphenols accumulation, Drought resistance

## Abstract

*Juglans regia*, an important economic tree species, is planted all over the world, and drought is one of the crucial factors limiting its growth and development. The various polyphenol content in walnut plants constitutes one of the material bases for the differences in stress resistance among various germplasms. However, the molecular mechanism underlying stress response mediated by polyphenol -dependent pathways remains unclear. v-Myb avian myeloblastosis viral oncogene homolog (MYB) protein of transcription factors play important regulatory roles in the process of plant stress responses. Previously, we identified *JrMYB44* could be involved in osmotic stress response in walnut. In this study, we confirmed that the drought resistance of four walnut cultivars (‘Chandler’, ‘Xiangling’, ‘Xilin2’ and ‘Xifu1’) is positively correlated with the accumulation of polyphenols. The content and component changes of polyphenols in *JrMYB44* overexpression (OE) and suppression (SE) lines in both walnut and *Arabidopsis thaliana* demonstrated that *JrMYB44* positively regulated polyphenols accumulation. The variation of *JrMYB44* expression and polyphenol levels under drought treatment indicated significant correlation between *JrMYB44*-induced drought tolerance and polyphenol accumulation, which was involved in reactive oxidative species (ROS) balance. The differentially expressed genes (DEGs) between OE and WT implied that *JrMYB44* could positively activate downstream genes to participate in the drought stress response. Yeast one-hybrid (Y1H), transient GUS expression assay and dual-luciferase reporter assay (DLR) confirmed that *JrMYB44* could recognize downstream *JrWRKY7* and *JrDREB2A*, two transcription factors previously reported to be involved in drought response. Meanwhile, it was confirmed by Y2H, GST-pull down and luciferase complementation imaging assay (LCI) that JrMYB44 could interact with JrMYC2 and JrDof1, another two previously reported potential drought response regulators. Collectively, these results indicated that JrMYB44 could activate *JrWRKY7*, *JrDREB2A* and interact with JrMYC2 and JrDof1 to promote walnut polyphenol accumulation and improve drought resistance in a ROS dependent manner.

**Supplementary Information:**

The online version contains supplementary material available at 10.1007/s44154-024-00193-7.

## Introduction

Walnut, as an important woody oil species, is widely planted all over the world mainly for providing nut and oil extraction (Bernard et al. 2021; Ding et al. 2022; Wambulwa MC 2022). In China, walnut is one of the main economic tree species in mountainous areas, and has been playing important roles in poverty alleviation and preventing reoccurrence (Yang et al. 2021). However, there were a lot of blind plantings using low-quality improved or mixed varieties, which resulted in the inability to ensure the yield and quality. Meanwhile, drought, as one of the key factors, restricts walnut growth and development (Wang et al. 2021a, b; Arab et al. 2022). In the arid areas of northwest China, the decline in groundwater level and the persistent water shortage have severely damaged the photosynthesis, plasma membrane system and osmotic potential adjustment, which leads to leaf shedding and even death, further restricting the development of walnut industry (Yang et al. 2022a, b). Therefore, it is necessary and urgent to reveal the mechanism of adversity adaptation in walnut production.

Grafting is the main propagation method in walnut cultivation. High-quality scion (the branch or bud) mainly determines fruit quality and yield, while excellent rootstock contributes to stress resistance for graft plants (Chilukamarri et al. 2021; Liu et al. 2023). Through grafting, the scion and rootstock are joined to achieve high resistance, excellent yield and quality, further promote the improvement of varieties, early earnings, and economic value (Ma et al. 2019). Evidently, effective evaluation of the stress response characteristics of scions and rootstocks, especially rootstocks, can predict the performance of nutrient transportation and normal growth and development under stress conditions, which will further promote the screening of excellent rootstocks and cultivate high-quality germplasm.

Polyphenols are secondary metabolites of plants, which have antioxidant effects both in vivo and in vitro. In response to abiotic stress, polyphenols could be mobilized to remove ROS accumulation and alleviate cell damage, which involved the inducement of stress resistance genes (Dini and Grumetto 2022; Shen et al. 2022). As a perennial Juglandaceae plant, walnut has abundant secondary substance both in kind and content that endowing it with strong antioxidant capacity for walnut (Fukuda et al. 2003). Previous studies have indicated that the component of polyphenols was related to cultivars (Pinasseau et al. 2017), and the content of polyphenols was higher in drought-tolerant genotypes than those in drought-sensitive ones (Juliano et al. 2019). We also found that the drought resistance of different walnut cultivars was related closely and intricately to secondary metabolites. The types and contents of polyphenols are also recognized as prominent criteria for comparing the stress resistance of different walnut cultivars. Nevertheless, to date, the relationship between the regulation of walnut polyphenol accumulation and drought adaptation mechanisms remains unclear, failing to adequately address the follow questions: Is the anti-stress effect of walnut related to the regulation of polyphenols? How do polyphenols regulate plant response to drought stress through related genes? In addition, the molecular mechanism of walnut polyphenol accumulation and metabolic regulation is still unclear, and the main genes have not been identified, which is not conducive to revealing the polyphenol metabolism pathway of walnut stress adaptation mechanism.

In other species, polyphenol synthesis has been reported to be involved in the MBW (MYB-BHLH-WD repeat) complex, where MYB is one of the families of plant transcription factors (TFs), containing a MYB domain at the N-terminal. MYB TFs play multiple regulatory roles in various physiological activities in plants, including growth and development, cell metabolism, cell morphology and pattern building (Timmermans et al. 1999; Pradhan et al. 2019; Wang et al. 2019a, b). Some *MYB*s can be induced by exogenous hormones such as jasmonic acid (JA) and ethylene, which can activate innate immunity in plants (Gu et al. 2019). However, the identification of *MYB*s in walnuts is far from sufficient, and it remains unclear how *MYB*s are involved in polyphenol accumulation and metabolism. Whether potential polyphenol regulation-related *MYB*s are related to stress responses such as drought has not been explored. In the previous study, we have identified 5 R2R3-MYBs from walnut. Among them, *JrMYB44* could be induced by PEG_6000_, and its upstream promoter contained abundant stress-related *cis*-acting elements. Meanwhile, *JrMYB44* functioned as an upstream regulator to control the downstream *JrGSTTau1* gene in osmotic stress response (Yang et al. 2019). Therefore, to clarify the association between polyphenol metabolism and drought resistance in walnut, in this study, the function mechanism of *JrMYB44* relating to polyphenols accumulation and drought stress response of walnut was explored. The results confirmed that the expression of *JrMYB44* is positive for walnut drought tolerance via polyphenols metabolism pathway.

## Results

### The content and component of polyphenols of four walnut cultivars

**Fig. 1:**
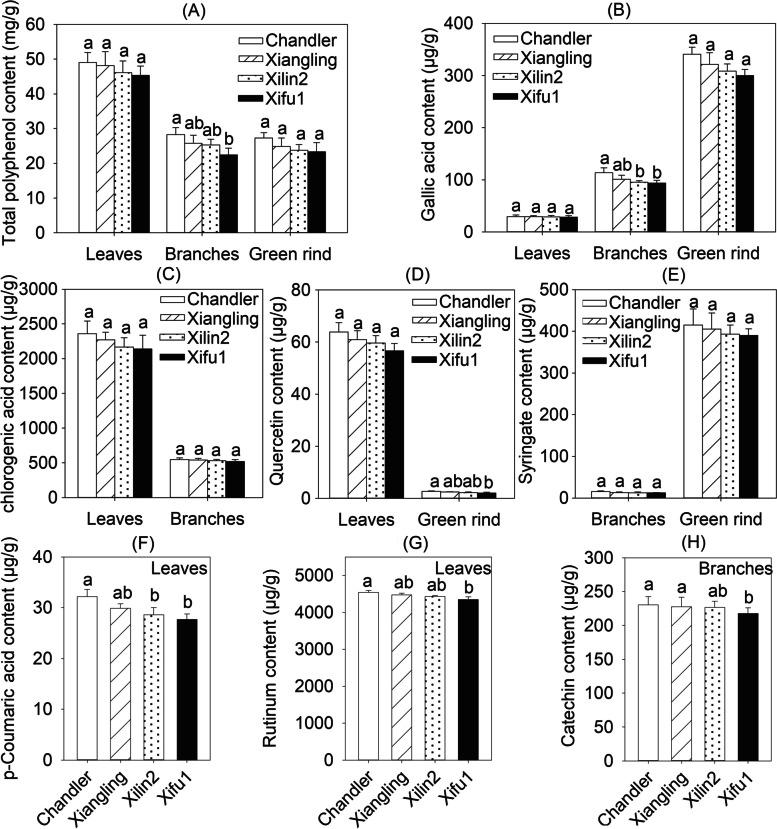
The polyphenols content and its components in the leaves, stems and green rinds from 7-year-old ‘Chandler’, ‘Xiangling’, ‘Xilin2’ and ‘Xifu1’. Differenct lowercase letters indicates significant differences among ‘Chandler’, ‘Xiangling’, ‘Xilin2’ and ‘Xifu1’ (*P* < 0.05). **A**, total polyphenol. **B**, gallic acid. **C**, chlorogenic acid. **D**, quercetin. **E**, syringate. **F**, p-Coumaric acid. **G**, rutinum. **H**, catechin. Each value represents the mean of three replicates and error bars represent the SD (*n* = 3) (The same below)

Four walnut cultivars (‘Chandler’, ‘Xiangling’, ‘Xilin2’ and ‘Xifu1’) with significant differences in drought stress tolerance were selected and their total polyphenols as well as the components were tested to verify the relationship of polyphenols with walnut drought stress response. The results showed that the total polyphenol contents in branches of four cultivars were significantly different (*P* < 0.05), and the maximum value was appeared in ‘Chandler’, whose total polyphenol content was 1.10 ~ 1.26-fold of ‘Xiangling’, ‘Xilin2’ and ‘Xifu1’. In leaf and green rind, the order of total polyphenol content from highest to lowest is still ‘Chandler’ > ‘Xiangling’ > ‘Xilin2’ > ‘Xifu1’ (Fig. 1A). The total polyphenol contents of the four cultivars were consistent with their drought resistance performance (Supplementary Fig. S1).

Further, the component of polyphenols of the leaf, branch and green rind were determined and found that some components displayed tissue specificity. The variation tendency of gallic acid in four cultivars was consistent with the total polyphenol (Fig. 1A-B). The contents of chlorogenic acid in leaves and branches as well as syringate in branches and green rind among the four cultivars were various not significantly (Fig. 1C, 1E). The quercetin content of ‘Chandler’ was 1.10 ~ 1.30-fold of others in green rind and the difference between ‘Chandler’ and ‘Xifu1’ was significant, while the changes in leaves were not obviously (Fig. 1D). The contents of leaf p-Coumaric acid were varied relatively large among the four cultivars, the maximum value of ‘Chandler’ is 1.16-fold of the minimum level appeared in ‘Xifu1’ (Fig. 1F). The contents of leaf rutinum and branch catechin were changed with similar profiles among the four cultivars that also the differences between ‘Chandler’ and ‘Xifu1’ were significant (Fig. 1G-H). The components of the polyphenols in different tissues were changed similar to the total polyphenols and some were significantly varied among ‘Chandler’, ‘Xiangling’, ‘Xilin2’ and ‘Xifu1’, suggesting the potential positive connection of polyphenol with walnut adaption to adverse stimulus. Especially p-Coumaric acid and rutinum in leaves, gallic acid and catechin in branches, quercetin in green rind deserve further attention.

### The regulation role of *JrMYB44* in polyphenols accumulation

We initially selected tissues same as differential polyphenols accumulation determination for transcriptome analysis to identify relevant genes. We found that one 849 bp R2R3-MYB (named *JrMYB44*) is more prominent. *JrMYB44* displayed strong transcription in old stems, as well as obvious induction exposing to 24 h PEG_6000_ stress (Yang et al. 2019; Li et al. 2021). In the drought resistance response of *JrGSTTau1*, *JrMYB44* acted as an upstream regulator (Yang et al. 2019). These preliminary results imply a close relationship of *JrMYB44* with drought stress response. Therefore, in this study, *JrMYB44* (accession No.: XP_018827388.1) was chosen for analysis. Here, the evolutionary relationship of *JrMYB44* was analyzed and showed that most of MYBs are related to polyphenol compound synthesis (such as AtMYB4, AtMYB75, PqMYB113, AtMYB15, PsMYB116, PsMYB37, PsMYB32, MaMYBPA1, MaMYBPA2, BraA07g032100.3C, BcaB05g24263, BcaB03g15272, AtMYB21, PpMYB44, AtMYB114, AtMYB90) and stress response (such as GaMYB85, AtMYB44, AtMYB70, AtMYB73, AtMYB49) (Supplementary Fig. S2, S3), further suggesting that *JrMYB44* may play potential roles in walnut drought response and polyphenol accumulation.

**Fig. 2:**
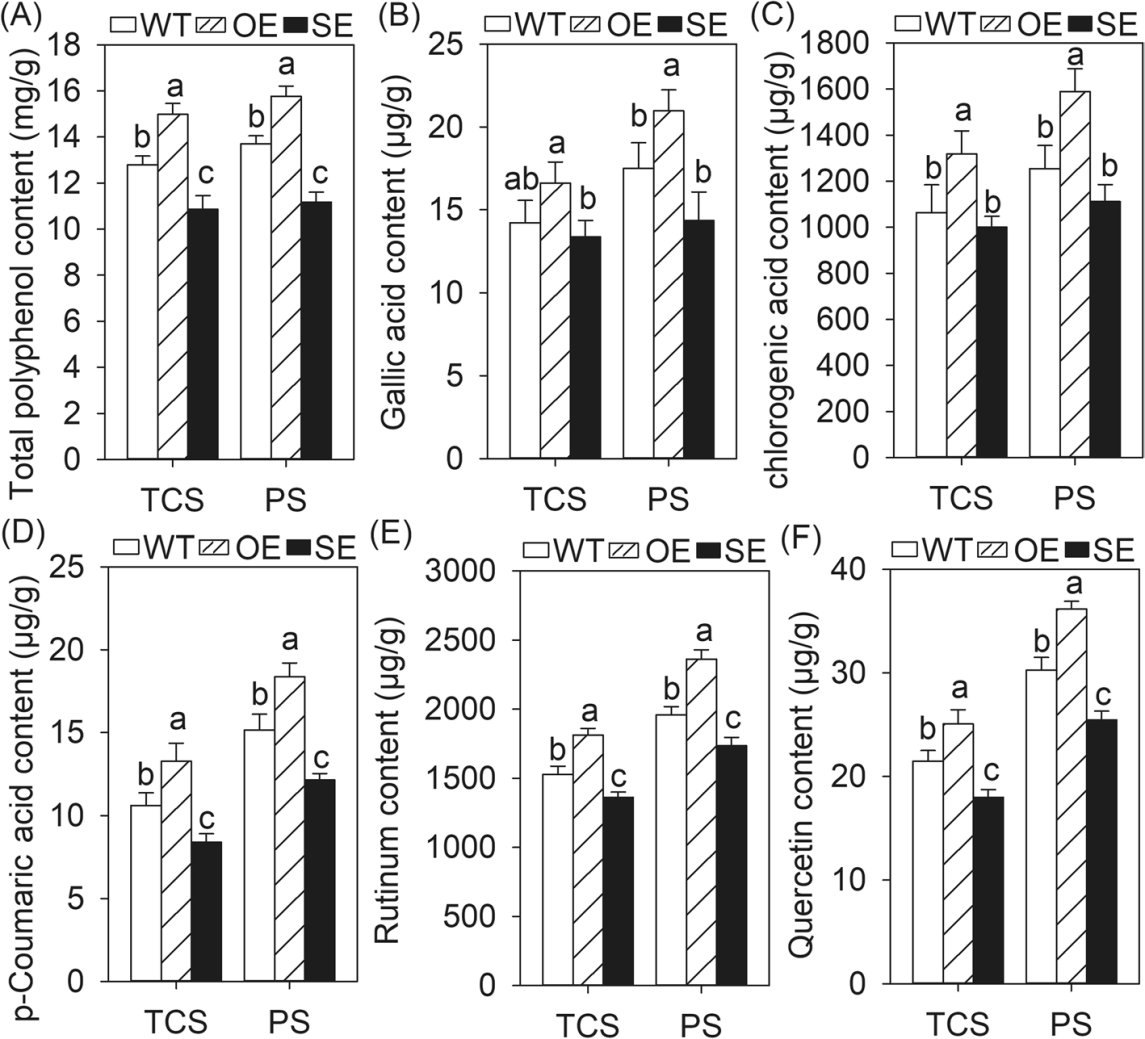
The content and components of polyphenols in the leaves of *JrMYB44* overexpression (OE) and suppression (SE) lines. TCS, tissue cultured sterile seedlings. PS, potted seedlings. Lowercase indicates significant differences among WT, OE, SE (*P* < 0.05). **A**, total polyphenol. **B**, gallic acid. **C**, chlorogenic acid. **D**, p-Coumaric acid. **E**, rutinum. **F**, quercetin

**Fig. 3:**
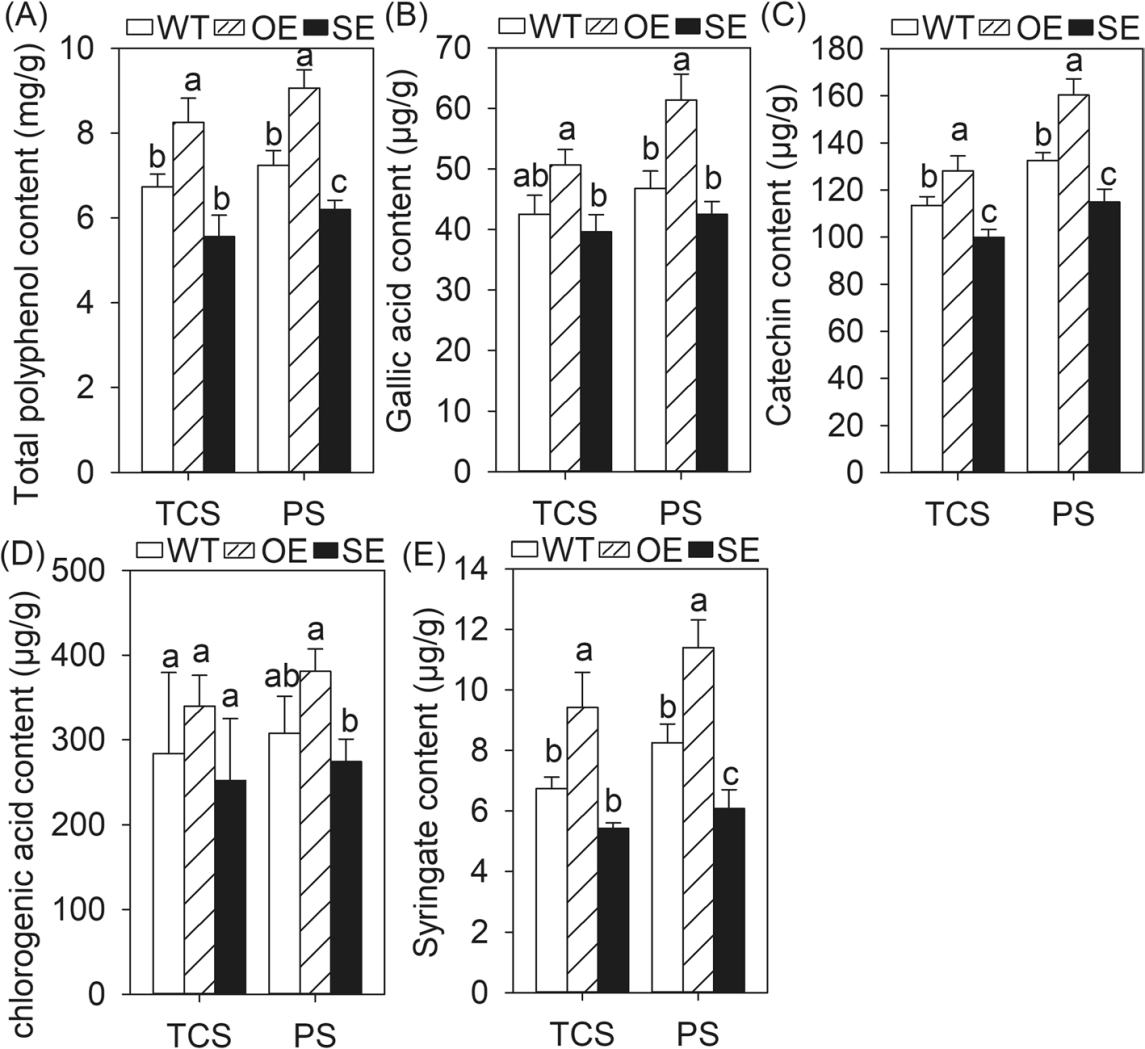
The content and components of polyphenols in the stems of *JrMYB44* overexpression (OE) and suppression (SE) lines. TCS, tissue cultured sterile seedlings. PS, potted seedlings. Lowercase indicates significant differences among WT, OE, SE (*P* < 0.05). **A**, total polyphenol. **B**, gallic acid. **C**, catechin. **D**, chlorogenic acid. **E**, syringate. Each value represents the mean of three replicates

*JrMYB44* overexpression lines (OEs) and suppression lines (SEs) were obtained to clarify whether *JrMYB44* is related to the accumulation of walnut polyphenols. Among the transgenic plants, the lines with expression levels exceeding 100 and inhibition levels below 30% were selected for subsequent analysis (Supplementary Fig. S4A, B). Then the total polyphenol content and related components in the leaves and stems of tissue cultured sterile seedlings (TCS) and potted seedlings (PS) (Supplementary Fig. S5) of WT, OE, and SE were measured (Fig. 2; Fig. 3). The result showed that the variation tendency of the content of total polyphenol and main components are consistent with the relative expression of *JrMYB44* both in leaves and stems. In detail, in leaves, the total polyphenol content varied significantly, and the content of OE reached 1.15 ~ 1.17-fold of WT, while SE was only 81.58% ~ 84.87% of WT (Fig. 2A). The content of gallic acid, chlorogenic acid, p-Coumaric acid, rutinum and quercetin were changed with similar profiles not only in TCS but also in PS. Compered to WT, these five were increased to 116.69% ~ 125.03% and 119.50% ~ 126.63% in OE while decreased to 79.14% ~ 94.02% and 80.10% ~ 88.62% in SE under TCS and PS conditions, accordingly (*P* < 0.05) (Fig. 2B-F). In stems, the total polyphenol content of OE in TCS and PS was 1.23 ~ 1.25-fold of WT (Fig. 3A). The gallic acid, catechin, chlorogenic acid and syringate of OEs were increased to 112.86% ~ 139.78% (TCS) and 121.02% ~ 138.00% (PS), respectively (Fig. 3B-E). The catechin deserve further attention with significantly varied in WT, OE and SE (*P* < 0.05). After suppression of *JrMYB44*, the total polyphenol and main components contents of SE were decreased in varying degrees. The total polyphenol content was down-regulated to 82.59% ~ 85.61% of WT (Fig. 3A). The gallic acid, catechin, chlorogenic acid and syringate acid were decreased by 6.85%-19.50% (TCS) and 9.13%-26.38% (PS), respectively (Fig. 3B-E). These results indicated that *JrMYB44* could positively regulate polyphenols accumulation in walnut.

*JrMYB44* was also transformed into *A. thaliana* to generate the OEs and SEs (Supplementary Fig. S4C, D). The contents of total polyphenol, and related components including chlorogenic acid, p-Coumaric acid, quercetin, catechin and syringate acid of aerial parts during bolting stage were determined (Supplementary Fig. S6). The results showed that, with the overexpression of *JrMYB44*, the total polyphenol content was increased significantly and 1.32 ~ 1.52-fold of WT (Supplementary Fig. S6A). The content of catechin, chlorogenic acid, syringate, p-Coumaric and quercetin was 1.12 ~ 2.14-fold of WT, and the p-Coumaric acid was changed most obviously in OE1 (Supplementary Fig. S6B-F). While introduced RNAi::*JrMYB44*, the expression of homologous similar protein *AtMYB44*, *AtMYB70* and *AtMYB73* was suppressed (Supplementary Fig. S3, S4E) and the select SE1 line displayed a 85% total polyphenol content of WT (Supplementary Fig. S6A). The catechin, chlorogenic acid, syringate, p-Coumaric and quercetin were 69% ~ 90% of WT (Supplementary Fig. S6B-F). These results indicated that *JrMYB44* could actively regulate the accumulation of polyphenol both in walnut and in *A. thaliana*.

### The relationship of *JrMYB44* with polyphenols accumulation under drought stress

**Fig. 4:**
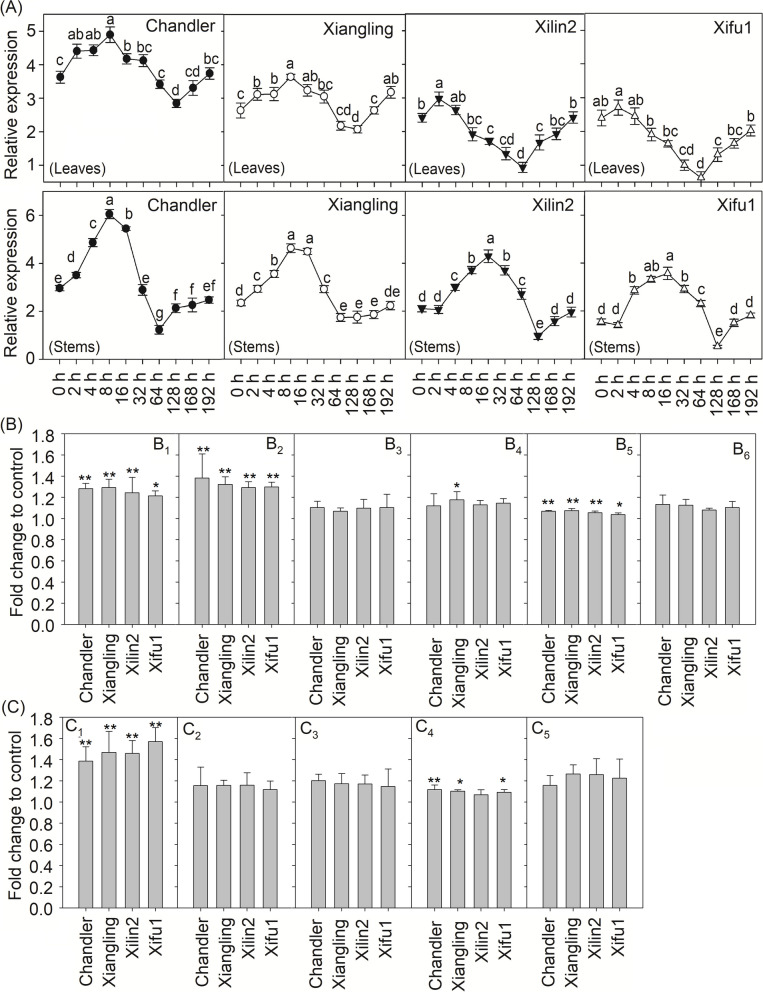
The expression of *JrMYB44* and the content and components of polyphenol in four walnut cultivars under drought stress. **A**, the relative expression of *JrMYB44* in leaves and stems under drought stress (20% (W/V) PEG_6000_ for 0, 2, 4, 8, 16, 32, 64, 128, 168, 192 h). The significant differences (*P* < 0.05) among different treatment time points were marked by lowercase letters. **B**-**C**, the content and components of polyphenols in leaves (**B**) and stems (**C**) under 0 (control) and 8 d of drought treatment and the results were figured according to control as fold-change. B_1_ and C_1_, total polyphenol. B_2_ and C_2_, gallic acid. B_3_ and C_3_, chlorogenic acid. B_4_, p-Coumaric acid. B_5_, rutinum. B_6_, quercetin. C_4_, catechin. C_5_, syringate. *, ** means the significant difference between 0 and 8 d of same index and cultivar at *P* < 0.05 and *P* < 0.01 level, accordingly

The expression levels of *JrMYB44*, as well as the total polyphenol content and key components, were analyzed in four cultivars under drought conditions to investigate their relationships. We found that under drought treatment, the relative expression trend of *JrMYB44* in four cultivars were similar, increased firstly and then decreased (Fig. 4A). In ‘Chandler’ and ‘Xiangling’, *JrMYB44* was reached the maximum at 8 h. Compared with untreated condition, the expression of *JrMYB44* was increased to 1.35-fold (leaf) and 2.04-fold (stem) in ‘Chandler’, 1.38-fold (leaf) and 1.97-fold (stem) in ‘Xiangling’, respectively. In ‘Xilin2’ and ‘Xifu1’, the maximum expression of *JrMYB44* in leaves and stems was appeared at 2 h and 16 h, respectively, whose expression was increased to 1.23-fold (‘Xilin2’), 1.13-fold (‘Xifu1’) in leaves, 2.05-fold (‘Xilin2’), 2.31-fold (‘Xifu1’) in stems of that under control condition, respectively (Fig. 4A).

After drought treatment, the contents of total polyphenols and related components were all increased in four cultivars (Fig. 4B, C). The total polyphenol was 1.21- ~ 1.29-fold (leaf) and 1.39- ~ 1.57-fold (stem) more compared to untreated ones (Fig. 4B1, C1). Among components, in leaves, gallic acid and rutinum were increased obviously (Fig. 4B2, B5). The content of gallic acid was 1.29- ~ 1.38-fold (leaf), and rutinum was 1.04- ~ 1.07-fold (leaf) of 0 h in four cultivars. Chlorogenic acid, p-Coumaric acid and quercetin were changed not significantly, excepting p-Coumaric in ‘Xiangling’ (Fig. 4B3, B4, B6). In stems, catechin was accumulated to 1.07- ~ 1.12-fold of that under control condition and the changes were significantly among cultivars excluding in ‘Xilin2’ (Fig. 4C4). In addition, the contents of gallic acid, chlorogenic acid and syringate were increased with varying degrees (Fig. 4C2, C3, C5). Correlation analysis found that the expression of *JrMYB44* in response to drought stress is significantly correlated with polyphenol accumulation (Fig. 4; Supplementary Fig. S1).

### The function of *JrMYB44* in response to drought stress

The *JrMYB44* overexpression and suppression lines were treated with drought stress and the growth and physiological related indicators were evaluated. There were no obvious differences among WT, OE and SE leaf phenotype before drought treatment; however, after drought stress, all leaves showed different degree of damage and SE was the most seriously (Supplementary Fig. S7A). Refer to water loss, OE has the slowest and least water loss, while SE has the fastest and most water loss (Supplementary Fig. S7B). EL rate and MDA content relating to cell damage and membranization degree of OE was 68.83% and 66.70% of that of WT; while SE was 1.34-, 1.15-fold of that of WT, accordingly (Supplementary Fig. S7C, E). These data told us that overexpression of *JrMYB44* reduced the degree of damage to walnut plants under drought stress.

**Fig. 5:**
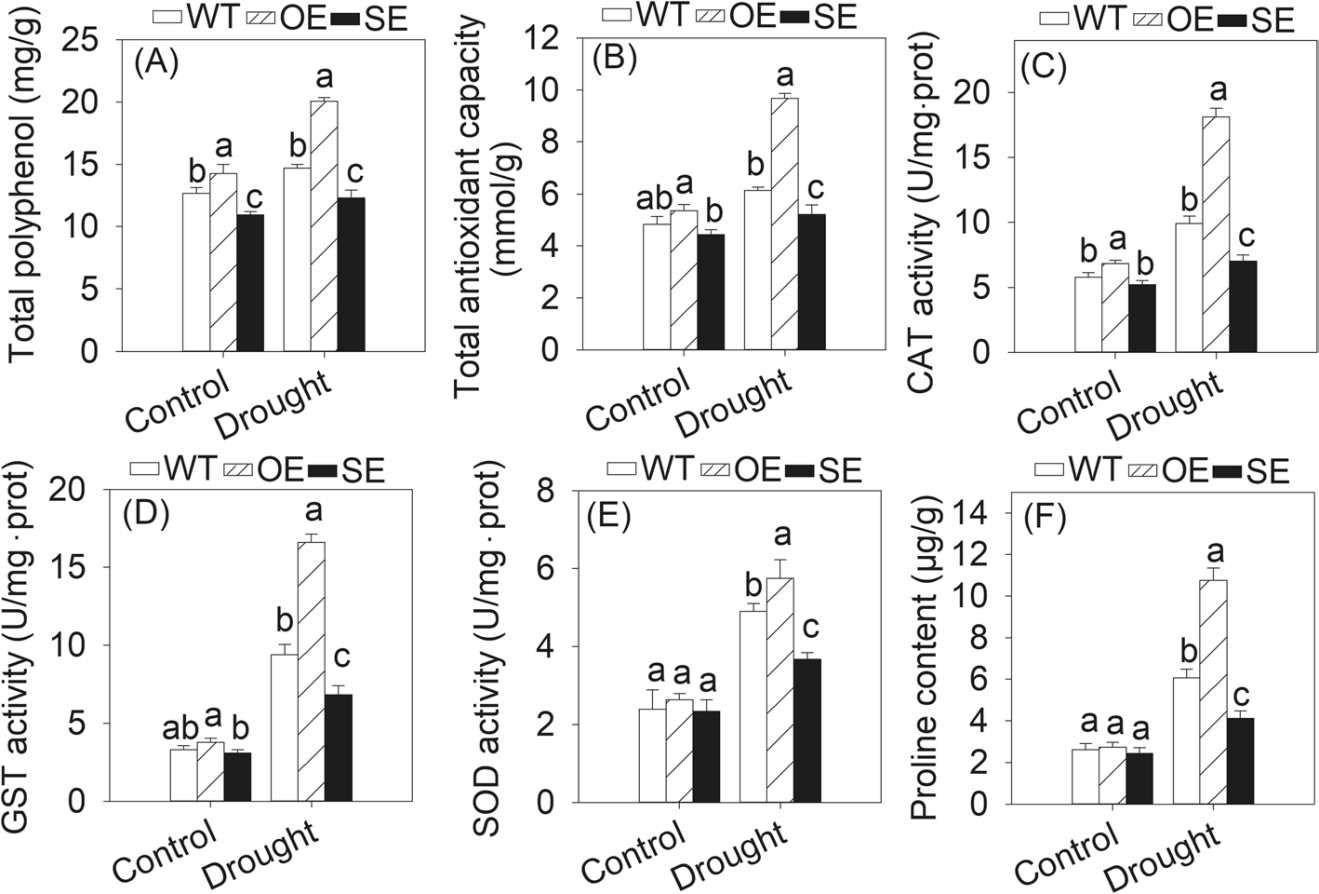
Polyphenol content and antioxidant capacity- related indexes in WT, transgenic walnut of OE (overexpression) and SE (suppression) lines. Lowercase indicates significant differences among WT, OE, SE (*P* < 0.05) under the same conditions. **A**, total polyphenol. **B**, total antioxidant capacity. **C**, CAT activity. **D**, GST activity. **E**, SOD activity. **F**, proline content

The tolerance to drought stress is often related to reactive oxygen species (ROS) scavenging. Testing the H_2_O_2_ content showed that SE accumulated 1.69-fold more ROS compared to WT (Supplementary Fig. S7D). However, the ROS scavenging related indexes were all reverse to ROS accumulation among OE, WT and SE. The total polyphenol content of OE leaves was significantly higher than that of WT (1.37-fold higher) and SE (1.63-fold higher). The total antioxidant capacity was 1.58-fold of WT and 1.86-fold of SE (Fig. 5A-B). The activities of catalase (CAT), glutathione S-transferase (GST) and superoxide dismutase (SOD) of OE were significantly improved than those of WT and SE after drought stress (Fig. 5C-E). Additionally, the proline content of OE reached 1.77-fold of WT and 2.61-fold of SE under drought stress (Fig. 5F). These results indicated that overexpression of *JrMYB44* could improve walnut resistance to drought stress involving cell health, ROS scavenging and proline metabolism, which was also confirmed in *Arabidopsis* with heterologous expression of *JrMYB44* (Supplementary Fig. S8).

### The downstream resistance genes recognized by *JrMYB44*

**Fig. 6:**
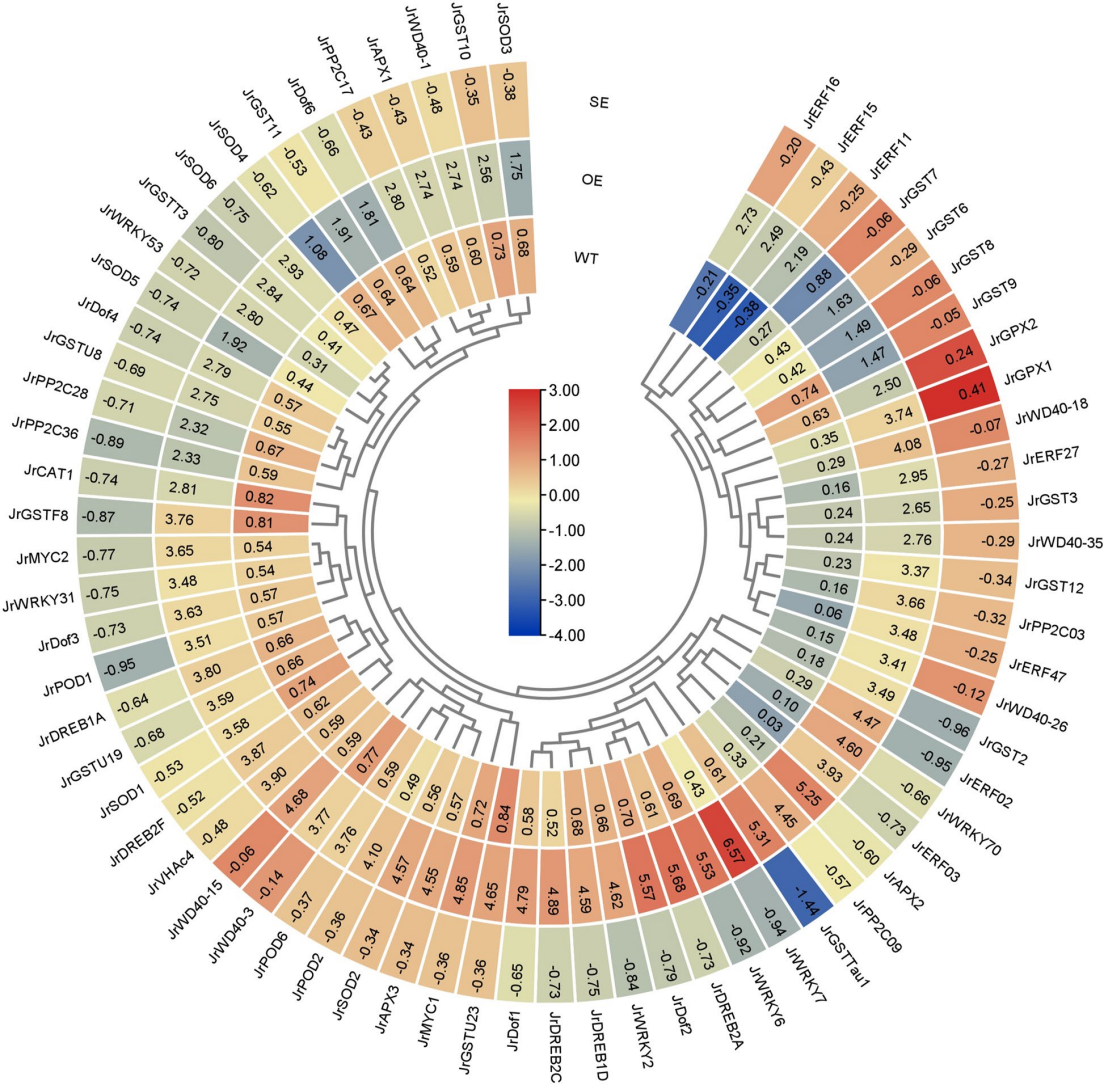
Clustering analysis of DEGs in walnut WT, OE and SE lines. The relative expression level was related to the expression of internal reference genes from three repeats. Heat map presented in blue/yellow/orange colors indicated low/medium/high expression, accordingly

The differentially expressed genes (DEGs) between OE and WT were determined and 4160 and 3753 DEGs were tested in leaves and stems, among which 2625 and 2112 were up-regulated, accordingly. GO analysis indicated that some biological processes, including ‘metabolic process’, ‘biological regulation’, ‘response to stimulus’, ‘signaling’ were significantly influenced by *JrMYB44*, and the verified genes were most selected from these biological processes. In molecular function clusters, the polyphenol accumulation related process (flavonoid, chitin, etc.) was also enriched. The expression levels of genes that are significantly up-regulated and probably involved in secondary substance metabolism in above GO subgroups were validated in OE, WT, and SE, and 76 genes were found to be affected by the expression of *JrMYB44* (Fig. 6, Supplementary Fig. S9). Among the 76 genes, 67 belong to the families of *GST, APX, GPX, CAT, SOD, POD, V-ATPase, WRKY, ERF, PP2C, DREB, MYC, DOF* and *WD40*. In OE, the *GSTs*, *DREBs*, and *WRKYs* were up-regulated to 2.00- (*JrGST9*) ~ 19.19-fold (*JrGST2*), 5.77- (*JrDREB1A*) ~ 9.49-fold (*JrDREB2C*), 6.46- (*JrWRKY31*) ~ 45.25-fold (*JrWRKY70*) of WT, accordingly. The other 9 genes, *PAL*, *CHS*, *F3H*, *F3'H*, *FLS*, *DFR*, *ANS*, *LDOX* and *ANR*, were related to polyphenol accumulation and transcribed to 2.61- ~ 5.25-fold of WT (Supplementary Fig. S9). However, in SE, the DEGs of *P5CS*, *PYL*, *ABF*, *SnRK* and *MAPK* families and nine polyphenol accumulation-related members were significantly down-regulated (Fig. 6, Supplementary Fig. S9). These expression performances of the downstream genes in OE and SE revealed that *JrMYB44* regulates walnut drought stress response and polyphenol accumulation via affecting the transcription of abundant downstream genes and pathways.

In addition, the expression levels of related DEGs were also detected in *JrMYB44* transgenic *Arabidopsis*. A total of 63 genes from *SnRK*, *PYL*, *ABF*, *DREB*, *SOD*, *P5CS*, *GST*, *APX*, *WRKY*, *PP2C*,* DOF* and *WD40* families were checked in WT, OE1, OE2 and SE1. We found that, compared to WT, *GSTs*, *DREBs*, *WRKYs* and other stress resistance related genes in OEs were consistently up-regulated, while they were down-regulated in SE1 (Supplementary Fig. S10). These changes further added to the evidence for conclusion that *JrMYB44* positively regulates drought stress response by actively mobilizing the expression of downstream stress-related genes.

### The recognition of *JrMYB44* to *JrWRKY7* and *JrDREB2A*

**Fig. 7:**
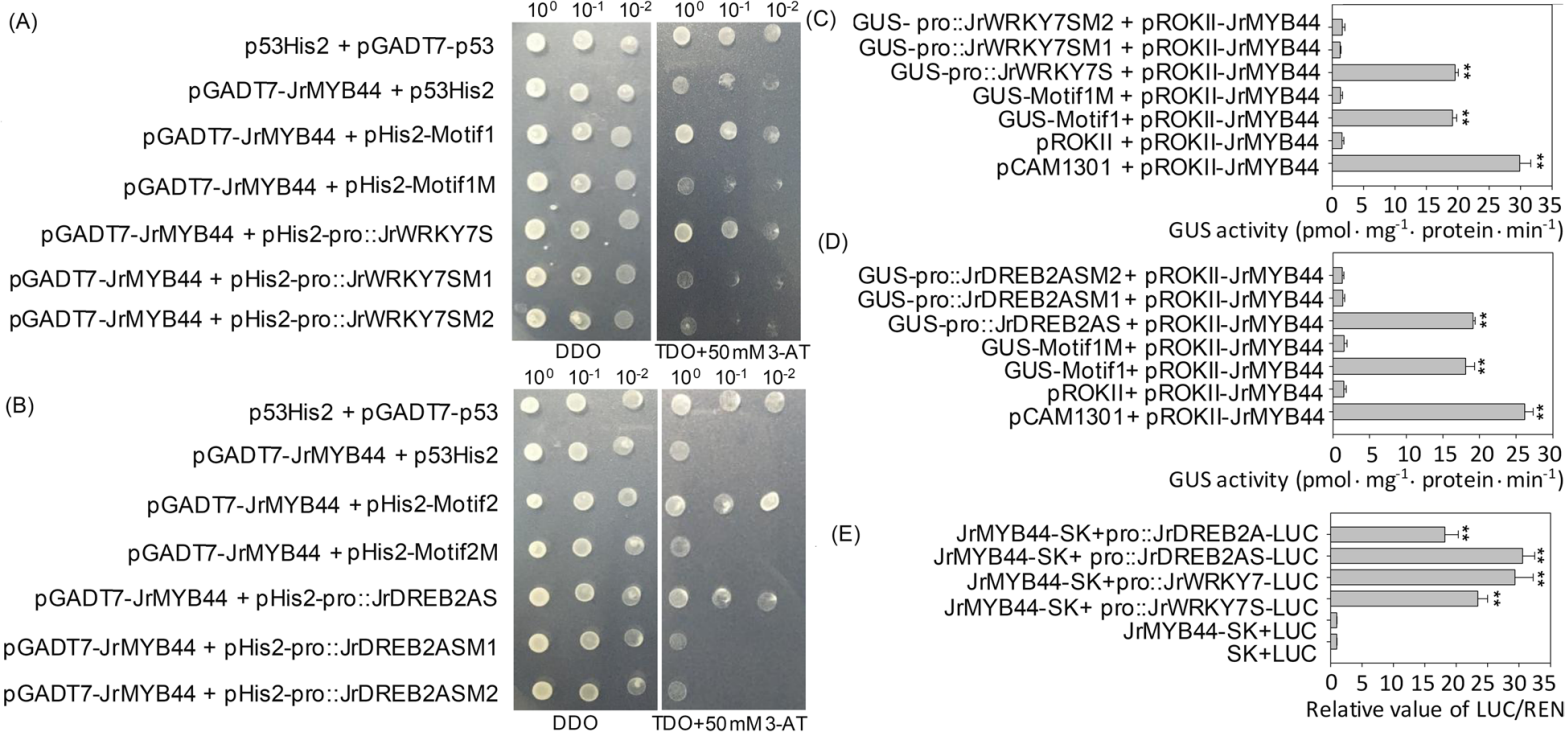
The recognition of *JrMYB44* to *JrWRKY7* and *JrDREB2A* by binding to their promoters. Motif1, MYBCOREATCYCB1 element in *JrWRKY7* promoter (sequence 'AACGG'). Motif2, MYBCORE element in *JrDREB2A* promoter (sequence 'CGGTTG'). Motif1M, mutated motif1 (‘GGACC’). Motif2M, mutated motif2 (‘ATTCCT’). pro::JrWRKY7S, pro::JrWRKY7SM1, pro::JrWRKY7SM2, the promoter segment containing the motif1, motif1M and excluding motif1. The same regular as motif2. **A**-**B**, Y1H assay. The transformants spotted on DDO were used as positive controls for transformant growth. Positive transformants were further confirmed by spotting serial dilutions (1/1, 1/10, 1/100) onto TDO plates added with 50 mM 3-AT. **C**-**D**, transient GUS expression assay. pROKII + pROKII-JrMYB44, negative control. pCAM1301 + pROKII-JrMYB44, positive control. **E**, DLR assay. The LUC:REN ratio of the negative control (SK + LUC) was set to 1. Values are presented as the mean ± SD based on three repetitions. ** indicates significant difference (*P* < 0.01) between each binding and the negative control

The expression of *JrMYB44* can change the transcriptional activities of many genes (including *GST*, *WRKY*, *DREB* and other families) (Fig. 6; Supplementary Fig. S9, S10), in which *JrWRKY7*, *JrDREB2A*, *JrMYC2* and *JrDof1* are the upstream regulators of *JrGSTTau1* in drought resistance response (Yang et al. 2019). Meanwhile, *JrWRKY7* and *JrDREB2A* were targeted the downstream of *JrMYB44* in a flavonoid synthesis and glutathione metabolism related pathway, accordingly (Supplementary Fig. S12). Therefore, to reveal the drought response mechanism, the possible relationship between *JrMYB44* and *JrWRKY7*, *JrDREB2A* were further analyzed. Firstly, the upstream promoters of *JrWRKY7* and *JrDREB2A* were checked to cover some MYB TFs recognition elements (Supplementary Fig. S11) and the recognition of *JrMYB44* to *JrWRKY7* and *JrDREB2A* were analyzed using yeast one-hybrid (Y1H) assay. The *JrWRKY7* promoter contained MYB TFs recognition elements like AMYBOX1, MYBGAHV, MYBCOREATCYCB1 (core sequence 'AACGG', represented by Motif1) (Supplementary Fig. S11A). A 237 bp promoter fragment containing Motif1 was selected for Y1H assay, and found that *JrMYB44* could bind to the Motif1 and the promoter segment containing Motif1, which was negatively proved by the interaction between pHis2-Motif1M (mutated Motif1), pHis2-pro::JrWRKY7SM1 (*JrWRKY7* promoter segment including mutated Motif1), and pHis2-pro::JrWRKY7SM2 (*JrWRKY7* promoter excluding Motif1) with *JrMYB44* on the SD (synthetic drop-out medium)/ − Trp-Leu-His/50 mM 3-AT (3-amino-1, 2, 4-triazole) solid medium (Fig. 7A). Moreover, the effective recognition of *JrMYB44* to *JrWRKY7* was further confirmed by GUS report system and dual-luciferase reporter (DLR) assay (Fig. 7C, E).

Similarly, we found four MYB TFs recognition elements in the 506 bp upstream promoter sequence of *JrDREB2A,* including MYB1AT, MYBST1 and MYBCORE (Supplementary Fig. S11B). A 171 bp promoter fragment containing MYBCORE (core sequence 'CGGTTG', represented by Motif2) was selected for Y1H assay and verified the recognition of *JrMYB44* to *JrDREB2A* (Fig. 7B), which was also further determined by GUS expression and DLR assay (Fig. 7D, E). Thus, above results revealed that *JrMYB44* could recognize *JrWRKY7* and *JrDREB2A* by binding with special motifs in corresponding promoter.

### The interaction between JrMYB44 with JrMYC2 and JrDof1

**Fig. 8:**
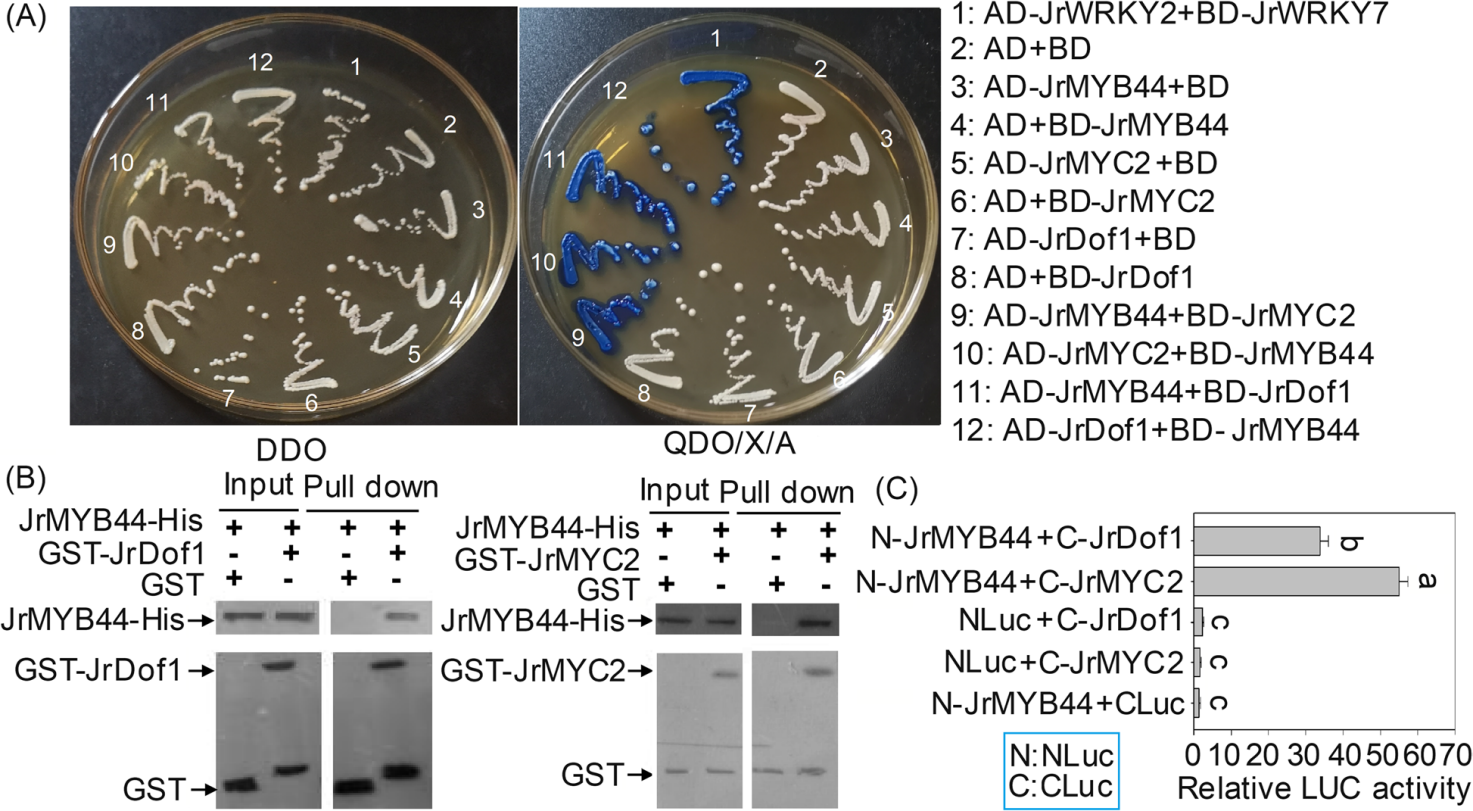
Interaction analysis of JrMYB44 with JrMYC2 and JrDof1. **A**, Y2H assay. AD + BD, AD-JrMYB44 + BD, AD + BD-JrMYB44, AD-JrMYC2 + BD, AD + BD-JrMYC2, AD-JrDof1 + BD, AD + BD-JrDof1 were used as negative controls. AD-JrWRKY2 + BD-JrWRKY7 was set as positive control. The DDO plates were growth control. QDO/X/A plats were interaction performance. **B**, In vitro GST-Pull down analysis of the interaction between JrMYB44 and JrDof1 as well as JrMYC2. Recombinant GST-JrDof1, GST-JrMYC2 and GST proteins were immobilized on glutathione agarose beads and incubated with JrMYB44-His protein. JrDof1 and JrMYC2 were detected with anti-His antibody. **C**, Luciferase complementation imaging (LCI) assays. Fusion proteins NLuc + CLuc-JrDof1, NLuc + CLuc-JrMYC2 and NLuc-JrMYB44 + CLuc was used as control. The fluorescence intensity was detected by luminometer. All assays were performed three times with similar results

Considering the co-location of JrMYB44, JrDof1 and JrMYC2 in the same modules of KEGG pathways involved in flavonoid synthesis and glutathione metabolism (Supplementary Fig. S12), the yeast two-hybrid (Y2H) assay was applied to analyze whether JrMYB44 could interact with JrMYC2 and JrDof1, respectively. It was found that either JrMYB44 as AD or BD could interact with JrMYC2 and JrDof1 to activate the expression of reporter genes and make the yeast grow normally with blue color on QDO/X/A solid medium which containing X-α-gal and Aureobasidin A (Fig. 8A). Furthermore, we performed GST-pull down assays using the recombinant proteins JrMYB44-His and GST-JrDof1, GST-JrMYC2 or GST alone. The results showed that JrMYB44-His was pulled down by GST-JrDof1 and GST-JrMYC2 but not by GST (Fig. 8B), suggesting that JrMYB44 could interact with JrDof1 and JrMYC2. We also fused JrMYB44 with NLuc (NLuc-JrMYB44, empty protein marked NLuc), JrDof1 and JrMYC2 with CLuc (CLuc-JrDof1 or CLuc-JrMYC2, empty protein marked CLuc), respectively, to demonstrate the interaction between JrMYB44 and JrDof1, JrMYB44 and JrMYC2. The results showed that the LUC activity in NLuc-JrMYB44 + CLuc-JrDof1 or CLuc-JrMYC2 was significantly higher than control (*P* < 0.05) (Fig. 8C). The above results fully indicated that JrMYB44 can interact with JrMYC2 and JrDof1.

## Discussion

Drought is one of the important limiting factors affecting walnut cultivation. Walnut plants have a high content of polyphenols and antioxidant activity, which are the material basis for walnuts to respond to stress such as drought. However, the molecular mechanism between the key genes regulating walnut polyphenols and drought response is not yet clear, which is not conducive to the effective development of walnut drought resistance cultivation, nor is it beneficial to the selection of drought resistant rootstocks and scions, which restricts the benefits of the walnut industry. Polyphenols are the most abundant secondary metabolites in plants. They regulate oxidative stress by chelating iron and scavenging reactive radicals, preventing or even inhibiting oxidation, and have strong antioxidant capacity (Dini and Grumetto 2022). In coping with biological and abiotic stresses, the accumulation of polyphenols plays an important role in counteracting the negative effects of stress on plants (Sharma et al. 2019; Dini and Grumetto 2022). Under drought stress, plants produce stress-induced ROS and then use ROS as signaling molecules to activate stress-tolerance mechanisms. The adaptation of walnuts to adversity is closely related to the abundance of polyphenols. Therefore, it is feasible to elucidate the molecular mechanism of walnut drought stress response based on polyphenols-dependent pathway.

In order to effectively identify the key factors associated with walnut polyphenol accumulation and drought resistance regulation, we have observed the drought resistance performance of nearly 20 different walnut germplasms for a long time and selected four cultivars ('Chandler', 'Xiangling', 'Xilin2', 'Xifu1') with significant differences (Liu et al. 2014) for polyphenol content and components determination. We found that the rich accumulation of walnut polyphenols is an important foundation for strong drought resistance (Fig. 1). This provides effective data support for determining the correlation between walnut polyphenols and drought resistance. To clarify the molecular regulatory mechanism of walnut polyphenol accumulation, based on the previous transcriptome data and research on the functional mechanism of drought resistance genes (Yang et al. 2019; Li et al. 2021), we identified a highly promising R2R3-MYB, *JrMYB44*, which shares a close evolutionary relationship with abundant homogenous proteins such as AtMYB75, AtMYB21, AtMYB4, StMYB44 (Supplementary Fig. S2), all of which were reported to be involved in secondary metabolite metabolism and stress response (Qi 2011; Kang et al. 2020; Wang et al. 2020; Zhang et al. 2021). However, it is still unclear whether these *MYB*s achieve drought stress response by regulating polyphenol accumulation. Here, we found that the expression activity of *JrMYB44* under drought stress is significantly correlated with polyphenol accumulation and component composition, and is also consistent with the differences in drought resistance among cultivars (Fig. 4; Supplementary Fig. S7).

Previous studies have shown that some plant R2R3-MYB TFs, such as *CsMYB5a*, *CsMYB5*, *GhMYB18*, *AtMYB5*, *AtMYB23*, could regulate the accumulation or metabolism of secondary biological substances (Feng et al. 2020; Hu et al. 2022; Jiao et al. 2023). Here, we demonstrate that the R2R3 MYB TF *JrMYB44* also positively regulates the abundance and components of walnut polyphenols. Transgenic walnut and *Arabidopsis* overexpressing *JrMYB44* were endowed with significantly increased polyphenol content and components while suppression lines were deprived of effective enrichment of polyphenols compared to WT plants (Fig. 2 ~ 3; Supplementary Fig. S6), a positive feedback of polyphenols regulation presented by *JrMYB44*. Interestingly, we observed that drought stress induced an increase in polyphenol accumulation in all plant lines (WT, OE, SE), but the increase in OE is more pronounced (Fig. 5A). Meanwhile, the expression of nine genes related to polyphenol accumulation in OE were up-regulated than that in WT, while they were significantly down-regulated with different degrees in SE (Supplementary Fig. S9). These performances indicated that the high-level expression of *JrMYB44* can also associate with the response to drought stress by regulating polyphenol abundance. Furthermore, under drought stress, compared to WT, OE exhibited better leaf phenotype, slower water loss efficiency, milder cell damage, less ROS accumulation, and higher antioxidant protective enzyme activity; while SE, on the other hand, was exactly the opposite of OE (Supplementary Fig. S7 ~ S8; Fig. 5), confirming that the expression of *JrMYB44* could promote the walnut tolerance to drought stress. Based on the above, we consider that *JrMYB44* achieves drought resistance adaptation in walnuts by regulating the accumulation of polyphenols.

Our results demonstrated that *JrMYB44* functions as a central regulator in drought stress response. Several pieces of evidences support this conclusion. First, *JrMYB44* affects the transcription of multiple genes involved in abiotic stress especially drought tolerance (Fig. 6, Supplementary Fig. S10). For instance, the vacuole H^+^-ATPase (*V-ATPase*) c subunit *JrVHAc4* and tau sub-type GST *JrGSTU23* were up-regulated in OE. Both *JrVHAc4* and *JrGSTU23* have been proved to play positive roles in drought stress (Yang et al. 2022a). Among many other up-regulated genes involved, the majority are related to antioxidant protection. For example, the members of *GST, APX, GPX, CAT, SOD, POD,* and *V-ATPase* families were widely verified to protect plants from oxidative stress caused by various environmental stimuli (Semra et al. 2015; Xu et al. 2017, 2018; Che et al. 2022; Yang et al. 2022a). Some of the TFs from *WRKY, ERF, PP2C*, and *WD40* were believed as vital candidates for drought stress response (Wang et al. 2021b; Yang et al. 2021; Chen et al. 2022a, b; Chen et al. 2023). *DREB* is a drought responsive element binding transcription factor family, widely recognized as a key regulatory gene in plant drought response (Mei et al. 2022). The *MYC* family also has an increasing number of members, of which have been proven to positively regulate plant drought tolerance (Sharma et al. 2023; Xu et al. 2023)*.* Therefore, the response of *JrMYB44* to drought stress may involve antioxidant protection pathways and multiple transcriptional regulations.

Second, *JrMYB44* was previously demonstrated to be an upstream regulator of *JrGSTTau1* same as *JrWRKY7* and *JrDREB2A* (Yang et al. 2019). In current study, according to the KEGG pathways based on the transcriptomes compared between *JrMYB44* overexpression lines and WT, *JrMYB44* appeared on the upstream of *JrWRKY7* and *JrDREB2A* (Supplementary Fig. S12). Moreover, we verified that *JrMYB44* could recognize MYBCOREATCYCB1 in *JrWRKY7* promoter and MYBCORE in *JrDREB2A* promoter (Supplementary Fig. S11; Fig. 7). MYBCORE like elements are typical stress response related ones that could be recognized by many MYB TFs in response to abundant stresses. Fore example, *BpMYB1* regulates the Cd up-take in paper mulberry (Xu et al. 2024), *OsCYP-25* respond to heat, cold, and drought in rice (Trivedi et al. 2014), and *BplMYB46* affects salt and drought tolerance in *Betula platyphylla* (Guo et al. 2016). Therefore, the recognition of *JrMYB44* to MYBCORE like elements further indicated the potential positive response to drought stress. Furthermore, *JrMYB44* was validated to recognize *JrWRKY7* and *JrDREB2A* as their common upstream regulator (Fig. 7, Supplementary Fig. S12B). The enhanced transcription of *JrMYB44* elevated the expression of *JrWRKY7* and *JrDREB2A* (Fig. 6), implying that in drought stress adaptation in walnut, *JrMYB44* may function as a higher-level regulatory factor compared to *JrWRKY7* and *JrDREB2A*. However, whether and how *JrMYB44* directly regulates *JrWRKY7* and *JrDREB2A* in concrete life activities needs further elucidation.

Third, *JrMYB44* could independently interact with JrMYC2 and JrDof1, which were confirmed using assays of Y2H, pull-down and luciferase complementation imaging assays (Fig. 8). *JrMYC2* and *JrDof1* could respond quickly to drought stress with tissue expression specificity, and could regulate the response of *JrGSTTau1* to drought stress (Yang et al. 2019). In the current study, we observed that the expression levels of *JrMYC2* and *JrDof1* were significantly enhanced in walnut plants overexpressing *JrMYB44*, while significantly repressed in walnut plants with *JrMYB44* inhibition (Fig. 6). The response of *JrMYB44, JrMYC2*, and *JrDof1* to drought stress is consistent. Meanwhile, *JrMYB44, JrMYC2*, and *JrDof1* were targeted in the same module of the KEGG pathways (Supplementary Fig. S12). Wang et al. pointed out that the R2R3-MYB AtMYB29 could interact with AtMYC3 by its C-terminal MYC-interaction motif, and MYB29-MYC3 binding model is widely applied among MYB-bHLH complexes in *Arabidopsis* (Wang et al. 2022). JrMYC2 shared close relationship with AtMYC3 with a 50.50% sequence similarity (Supplementary Fig. S13A). Both *JrMYB44* and AtMYB29 belong to R2R3-MYB, and with a similarity of 46.93% (Supplementary Fig. S13B), implying that the MYB44-MYC2 binding model may also exist widely in walnut. There are few reports on the interaction between TFs of MYB and Dof, and the published interactions between MYB and Dof mainly focus on barley seed development (Diaz et al., 2002; Diaz et al. 2005). The interaction between *JrMYB44* and JrDof1 discovered here expanded the MYB-Dof binding model with novelty. According to above findings, we also have reason to believe that the interactions between *JrMYB44*, JrMYC2, and JrDof1 will promote drought tolerance in walnuts.

## Conclusions

**Fig. 9:**
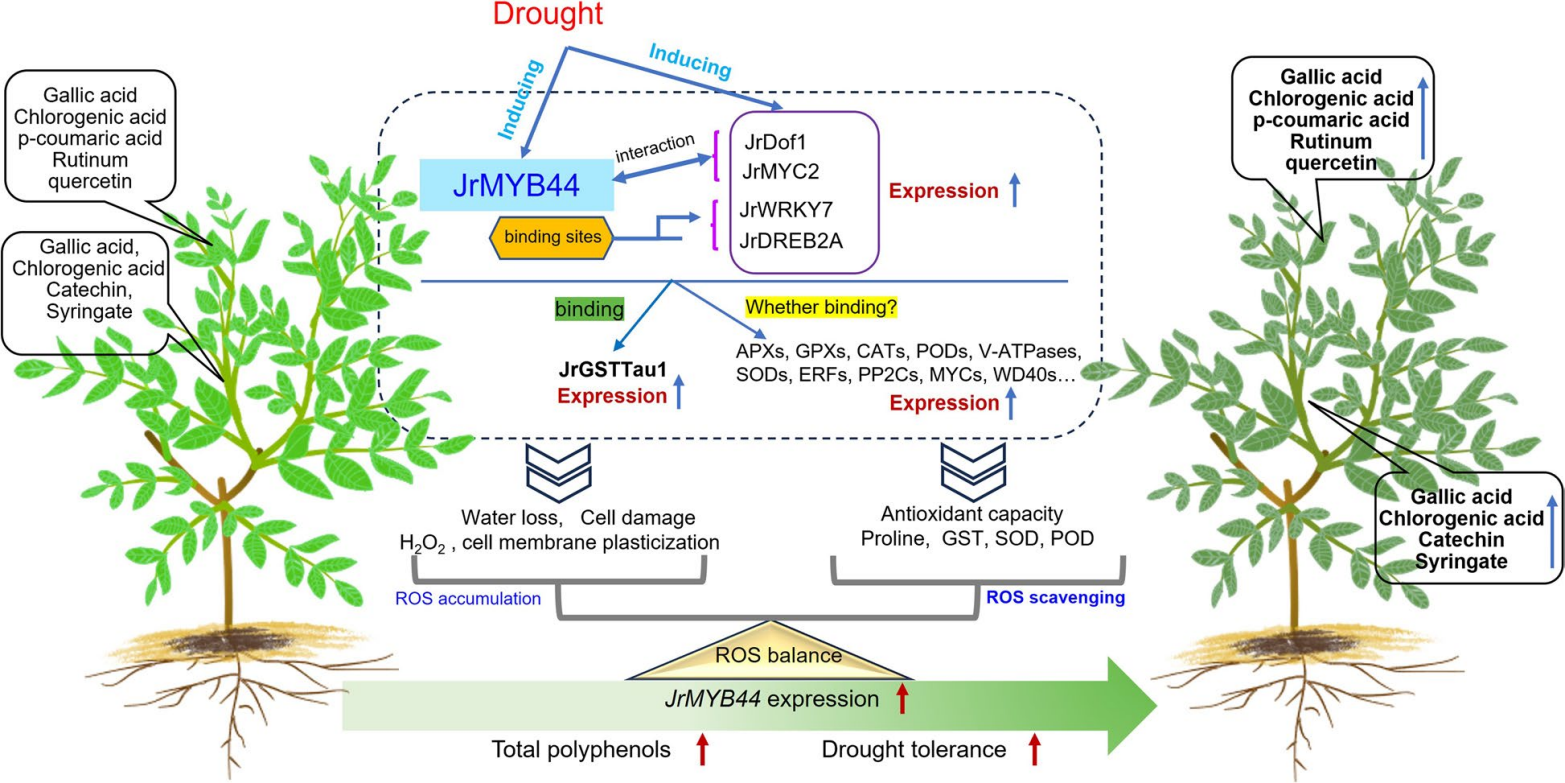
Model of *JrMYB44*-mediated signaling in response to drought stress involving polyphenol accumulation in walnuts. The induction of *JrMYB44* plays a significant role in enhancing walnut drought tolerance and total polyphenol accumulation. Specifically, *JrMYB44* engages in the interactions with *JrMYC2* and *JrDof1*, simultaneously regulates downstream target genes like *JrWRKY7* and *JrDREB2A*, thereby effectively maintaining the ROS balance. Via this mechanism, *JrMYB44* contributes to the drought tolerance and polyphenol enrichment in walnuts

Our findings demonstrated that *JrMYB44* positively regulates walnut polyphenols accumulation and drought tolerance. It achieves this by directly regulating *JrWRKY7* and *JrDREB2A* as well as interacting with JrMYC2 and JrDof1 involving antioxidant protection and ROS scavenging. This leads to the formation of a feedback mechanism based on recognition between *JrMYB44* and downstream targets and interactions between JrMYB44 and binding proteins related to polyphenols enrichment and drought tolerance (Fig. 9). The results of this study have enabled us to comprehend the mechanisms underlying the plant response to drought stress through the central action of *JrMYB44*. Moreover, considering that the content and components of walnut polyphenols are correlated with the expression level of *JrMYB44*, and that the drought resistance performance of different walnut germplasms is consistent with the transcriptional activity of *JrMYB44*, we propose a strategy for selecting drought resistant walnut germplasm based on *JrMYB44* expression and a pathway for creating excellent rootstocks or scions based on the overexpression of *JrMYB44*.

## Materials and methods

### Plant materials and treatments

Four walnut cultivars, ‘Chandler’, ‘Xiangling’, ‘Xilin2’, and ‘Xifu1’ with obvious difference in drought tolerance (Liu et al. 2014), planted at Shanyang Walnut Experimental Demonstration Station (Shangluo, China) were selected to determine the polyphenol content. The leaves, branches and green rinds from 7-year-old walnut cultivars propagated by grafted were sampled independently with three repeats. For expression analysis of *JrMYB44*, 3-year-old grafting seedling of the four cultivars were potted cultivated in a greenhouse (22 ± 2 °C, 70 ± 5% relative humidity, 14/10 h light/dark). 20% (W/V) PEG_6000_ was watered to the roots of the seedlings, then the leaves and stems were collected at 0, 2, 4, 8, 16, 32, 64, 128, 168, 192 h and saved in -80 °C refrigerator for further RNA isolation and polyphenol determination. The treatment was repeated three times and each repeat contained 5 seedlings.

### Determination of the content of polyphenols and their components

The walnut tissue sample was ground to powder by adding liquid nitrogen and screened with a pore size of 0.25 mm. The total polyphenol was determined by the Folin-phenol method (Rosa et al., 2010). 0.2 g of the powder was mixed with the extraction solution to obtain polyphenols. For the leaf sample, the material to extraction solution (M-ES) ratio was 1:40, the processing time (PTi) was 20 min, the ethanol concentration (EC) was 70%, the processing temperature (PTe) was 60 ℃, and the ultrasound power (ULP) was 60%. For the branches, the corresponding M-ES ratio was 1:60, PTi was 20 min, EC was 70%, PTe was 60℃, and ULP was 50%. For the green grind, the corresponding M-ES ratio was 1:40, PTi was 20 min, EC was 30%, PTe was 60℃, and ULP was 60%. The equipment used for ultrasonic processing is a bath type CNC ultrasonic equipment. After extraction, the mixture was centrifuged for 5 min with 8000 rpm to obtain the supernatant as the extraction solution, from which 1 mL was taken into a test tube and distilled water was added for a certain proportion of dilution. Then 1 mL of the dilution solution was used to measure the absorbance according to the standard curve and calculate the yield of polyphenols, which was expressed by extract Gallic acid equivalent (GAE mg·g^−1^ FW).

For analysis of components of the polyphenols by High Performance Liquid Chromatography (HPLC), 3.5 g sample powder sieved through a 0.25 mm pore size sieve was taken into a 250 mL conical flask and added by 100 mL of 50% methanol aqueous solution, which was placed in a bath type CNC ultrasonic equipment to extract for 30 min at 50℃ and 300 W twice. The extract was centrifuged at 8000 rpm for 10 min, and the supernatant was concentrated under reduced pressure at 65℃ to a paste shape. The supernatant was then diluted with 50% methanol to 5 mL to obtain the polyphenol extract, which was filtered using a 0.22 μM hydrophilic membrane and placed in a 1.5 mL injection bottle. For detection, the chromatographic column is CAPCELLPAKC18, with a mobile phase of 0.8% acetic acid solution (phase A) and chromatographic methanol (phase B). The injection volume is 5 μL with a flow rate 0.9 mL/min. The reference substances included gallic acid, catechins, chlorogenic acid, vanillic acid, caffeic acid, syringic acid, epicatechin, syringal, coumarin, ferulic acid, rutin, myricetin, and quercetin.

### RNA isolation, cDNA synthesis and RT-qPCR assay

The total RNA of each sample was isolated using cetyltrimethylammonium ammonium bromide (CTAB) method and digested by DNase. The total volume of RNA used was 1 μg in reaction system for cDNA reverse-transcription. The purified RNA was reverse-transcribed into cDNA using PrimeScript^TM^RT reagent Kit (CWBIO, Beijing, China). The cDNA was diluted to 1/10 and used as the template of RT-qPCR, which was implemented using the SYBR Green Real time PCR Master mix (CWBIO) and carried out on a CFX96 Touch™ Real-Time PCR Detection System (Bio-Rad Laboratories, Redmond, WA). The reaction parameters were: 94℃/30 s; 44 cycles of 94℃/12 s, 60℃/30 s, 72℃/45 s; 81℃/1 s. Each cDNA was used to apply three independent 20 μL reaction mixture to ensure the reproducibility of RT-qPCR results. The *18S rRNA* (HE574850) (Xu et al. 2012) and *GAPDH* (Czechowski et al. 2005) were used as internal control genes. The relative expression was calculated according to 2^−ΔΔCT^ method (Livak and Schmittgen 2001). All the primers were displayed in Supplementary Table S1.

### Basic bio-information and promoter analysis

The cDNA sequence of *JrMYB44*, *JrWRKY7*, *JrDREB2A*, *JrMYC2*, and *JrDof1* was obtained from walnut transcriptome and PCR amplified using primers for gene cloning (Supplementary Table S2). The conserved domain of each protein was predicted by the online tool ‘Conserved Domain Search Service (CD Search)’ (https://www.ncbi.nlm.nih.gov/Structure/cdd/wrpsb.cgi). The homologous sequences were found in related literatures (Qi, 2011; Butt et al. 2017; Chen et al. 2019; Wang et al. 2019a, b; Kang et al. 2020; Kim et al. 2020; Wang et al. 2020; Zhang et al. 2020; Liu et al. 2021; Zhang et al. 2021; Chen et al. 2022a; Cheng et al. 2022; Rajput et al. 2022; Yang et al. 2022b) and downloaded from NCBI (https://jgi.doe.gov/), TAIR (https://www.arabidopsis.org/) and JGI (https://jgi.doe.gov/). MEGA7 was used for multiple amino acid sequence alignment and evolutionary relationship analysis by constructing neighbor-joining tree (bootstrap = 1000). Online iTOL (https://itol.embl.de/) was used for phylogeny tree visualization. TBtools was used for making gene expression heat map. The sequences similarity was determined using blastp of NCBI. The promoters of *JrWRKY7* and *JrDREB2A* were obtained from walnut genome (Marrano et al. 2020) and PCR amplified using walnut DNA as template. The elements in the promoters were analyzed using New PLACE (https://www.dna.affrc.go.jp/PLACE/?action=newplace) and PlantCARE (https://bioinformatics.psb.ugent.be/webtools/plantcare/html/).

### Generation of plants overexpression or suppression of *JrMYB44*

The full CDS of *JrMYB44* was cloned into pROKII vector under the control of the 35S promoter (pROKII-JrMYB44) and used to obtain overexpression (OE) transgenic lines. The RNAi silence expression vector (RNAi::JrMYB44) was constructed (Yang et al. 2016) to obtain suppression (SE) transgenic lines. The tissue culture seedlings of walnut were obtained from the stems of the well growing plants, and the genetic transformation system of walnut tree was established. pROKII-JrMYB44 and RNAi::JrMYB44 were each transformed into *Agrobacterium tumefacien*s EHA105 cells. The EHA105 was cultivated to OD_600_ = 1.0 ~ 1.2 and then diluted to OD_600_ = 0.1 ~ 0.3 used as the infection solution. The walnut leaves with petioles or tender stems were placed in the infection solution for 3 ~ 5 min and transferred to the cocultivation medium (1/2MS + 2.5% [w/v] sucrose) for 3 ~ 5 d in dark after surfacely absorbed using absorbent paper. After completing coculture, the infection explants were subjected to form callus on the differentiation selection medium (1/2MS + 0.05 mg/L NAA + 0.5 mg/L 6-BA + 2.2% [w/v] sucrose + 35 mg/L kanamycin, pH = 5.8 ~ 6.0). The antibiotic-resistant callus was applied to stem extraction medium (1/2MS + 0.1 mg/L NAA + 0.02 mg/L 6-BA + 2.2% [w/v] sucrose + 35 mg/L kanamycin, pH = 5.8 ~ 6.0) for bud differentiation and the adventitious buds were used for rooting. The kanamycin-resistant potential transgenic seedlings were verified by PCR and RT-qPCR analysis. Transgenic lines with highest expression level of *JrMYB44* (OE), and lowest expression of *JrMYB44* (SE) were chosen for further analysis. The classic dipping flower method was adopted for *Arabidopsis* transformation. The related primers were listed in Supplementary Table S2.

### Growth and physiological performance evaluation

The similar shoots of WT, OE and SE were independently inserted into 1/2MS for 30 days and used for determination of polyphenols and their components under the tissue culture condition. 30-d-old seedlings of WT, OE and SE were transplanted into soil pots for another four months and applied to evaluate the polyphenols accumulation and drought stress response. The seedlings were treated with 10% PEG_6000_ for 12 d. The same period plants watered normally were used as control. The phenotype was observed and leaves as well as stems were collected for determination of physiological indicators. Each condition was set three repeats, and each repeat include 10 ~ 12 seedlings. The weight of the leaves was measured at 0, 2, 4, 6, 8, 10, and 12 h after they have detached from the plant, then the water loss rate expressed as the varied weight from the tested time point to 0 h divided the weight at 0 h. Electrolyte leakage (EL) rate, proline content and SOD activity were determined according to our previous reports(Yang et al. 2019; Li et al. 2021). H_2_O_2_ content, CAT activity, GST activity and total antioxidant capacity were tested according to the instructions of Hydrogen Peroxide assay kit (Colorimetric method, A064-1–1), Catalase (CAT) assay kit (Visible light, A007-1–1), Glutathione S-transferase (GSH-ST) assay kit (Colorimetric method, A004-1–1), as well as total antioxidant capacity (T-AOC) determination kit (ABTS method, A015-2–1), accordingly. All these kits were bought from Nanjing Jiancheng Bioengineering Institute (http://www.njjcbio.com/). In transgenic *Arabidopsis*, the 35-d-old seedlings of WT, OE1, OE2 and SE1 were used. Each replicate contained 20 seedlings. The leaves were also stained with nitro blue tetrazolium (NBT) to evaluate the in vivo accumulation of O^2−^.

### Identification of *JrMYB44* downstream target genes

The DEGs between *JrMYB44* overexpression plants and WT were screened by comparison the transcriptomes of WT and OE to identified the potential targets of *JrMYB44*. The transcriptomes were sequenced and annotated by Biomarker (http://www.biomarker.com.cn). The genes that displayed superior expression level were further submitted to compare the transcription activity in 4-month-old WT, OE and SE under 10% PEG_6000_ treatment. The prominently expressed genes were concerned that their promoters were identified and the recognitions by *JrMYB44* were confirmed using Y1H, GUS expression and LCI assays. The expression was analyzed by RT-qPCR. All the related primers were listed in Supplementary Table S1. In transgenic *Arabidopsis*, the stress resistance related genes were also selected to understand the downstream targets of *JrMYB44*. All the primers were showed in Supplementary Table S3.

### Y1H assay

The Y1H assay was applied following the Matchmaker Gold Yeast One-Hybrid Library Screening System (Clontech). The CDS of *JrMYB44* was inserted into vector pGADT7_Rec2 (AD) and used as the effecter. Three tandem copies of MYBCOREATCYCB1 (core sequence ‘AACGG’, motif1), MYBCORE (core sequence ‘CGGTTG’, motif2), mutated motif1 (‘GGACC’), mutated motif2 (‘ATTCCT’) as well as the promoter segments (Supplementary Fig. S11) were independently inserted into vector pHis2 (BD) and used as reporters. Each reporter was co-transformed with the effecter into Y187 and grown on agar medium plates of DDO (SD/-Trp/-His) and TDO (SD/-Trp/-His/-Leu) supplemented with 50 mM 3-AT (3-amino-1, 2, 4-triazole) for 3 ~ 5 d at 30℃. The related primers are listed in Supplementary Table S2.

### Transient GUS expression assay

Three tandem copies of Motif1, Motif1M, Motif2, Motif2M, the promoter segments containing Motif1, Motif2, Motif1M, Motif2M and the promoter segments excluding Motif1 and Motif2 were respectively fused with a CaMV35S-46 minimal promoter and cloned into pCAMBIA1301 to drive GUS gene expression and function as reporters. pROKII-JrMYB44 was used as the effector. Each reporter was transient co-transformed with the effector into tobacco leaves based on *A. tumefaciens* mediated transformation method. Then the GUS activity was determined according to the previous reports (Jefferson 1989; Yang et al. 2017). All experiments were performed three times.

### Dual-Luciferase reporter (DLR) assay

The DLR assay was implemented according to the methods reported by Yang et al (2022b) with minor adjustment. The CDS of *JrMYB44* was inserted into pSupre1300 to generate the recombinant effector. The promoter segments of *JrWRKY7* and *JrDREB2A* were respectively cloned into pGreenII0800-LUC to form promoter reporting vectors. The effector and each reporter were transformed into *A. tumefaciens*, respectively. Then according to the *A. tumefaciens* mediated transient transformation method, the effector and reporter were mixed in equal amounts and injected into tobacco leaves and incubated under dark for 36 ~ 48 h. The Dual Luciferase Reporter Gene Assay Kit (Biyuntian, RG027, http://www.beyotime.com) was applied to determine the luciferase activity.

### Y2H assay

The full CDS of *JrMYB44*, *JrMYC2* and *JrDof1* were each cloned into the vector pGADT7_Rec (AD) to generate the recombinant ADs (AD-JrMYB44, AD-JrMYC2, AD-JrDof1), and inserted into vector pGBKT7 (BD), respectively, to generate the recombinant BDs (BD-JrMYB44, BD-JrMYC2, BD-JrDof1). Each AD and BD were co-transformed into the yeast to examine the potential interaction on the QDO (SD/-Ade/-His/-Leu/-Trp) agar medium plus with X-α-Gal and Aureobasidin A. The interaction between empty AD and BD was used as negative control, while the published interaction between JrWRKY2 and JrWRKY7 (Yang et al. 2017) was function as positive control. All related primers were listed in Supplementary Table S2.

### Pull down assay

The full-length CDS of *JrMYB44*, *JrDof1* and *JrMYC2* were cloned into the vectors of pET30a and pGEX4T-1, respectively, which were further introduced into Rosetta (DE3) for expression of JrMYB44-His, GST-JrDof1 and GST-JrMYC2 those induced by 0.1 mM isopropyl-b-thiogalactopyranoside. Soluble GST or GST-JrDof1 and GST-JrMYC2 were extracted and immobilized using a glutathione HiCap matrix (Qiagen). JrMYB44-His was incubated with immobilized GST or GST-JrDof1 and GST-JrMYC2, and the interaction was detected by western blotting analysis applying an anti-His antibody (Sigma-Aldrich). The related primers are displayed in Supplementary Table S2.

### LCI assay

The LCI assay was applied according to the methods reported by Chen et al. (2008). The CDS of *JrMYB44* was inserted into pROKII-NLuc to form the recombinant NLuc-JrMYB44 (empty vector marked as NLuc). The CDS of *JrMYC2* and *JrDof1* were independently cloned into pROKII-CLuc to form the recombinants CLuc-JrMYC2 and CLuc-JrDof1 (empty vector marked as CLuc). Then each NLuc and CLuc was transformed into *Agrobacterium* and injected into tobacco leaves. After 24 ~ 48 h infiltration, the luciferin was added, and the fluorescence intensity was detected by luminometer. Three replicates were completed and similar results were confirmed.

### Statistical analysis

All of data were analyzed using the Statistical Package for Social Science (SPSS) (SPSS, Chicago, Illinois), and all data were the average of three replicates. Tukey's multiple comparison test was used to evaluate the differences between the transgenic lines and WT, and the significance level was set at *P* < 0.05.

## Supplementary Information

Additional file 1: Supplementary Table S1 The primers used for RT-qPCR analysis in walnut. Supplementary Table S2 The primers used for construction of recombinant vectors. Supplementary Table S3 The primers used for RT-qPCR analysis in Arabidopsis. Supplementary Fig. S1 Correlation analysis of drought resistance, *JrMYB44* expression and polyphenols. *, **, and *** indicates significant correlation at *P*<0.05, *P*<0.01 and *P*<0.001 level, accordingly. Supplementary Fig. S2 Phylogenetic tree analysis of JrMYB44 protein and its homologs from other species based on sequence alignments of the encoded proteins using neighbor-joining method in MEGA7. Jr, *Juglans regia*; At, *Arabidopsis thaliana*; Ga, *Gossypium arboretum*; Ma, *Musa acuminata*; Ptr, *Populus trichocarpa*; Ps, *Pisum sativum*; Pq, *Paeonia qiui*; BraA07g032100.3C is a MYB of *Brassica rapa*, BcaB05g24263 and BcaB03g15272 are MYBs of *B. carinata*; St, *Senna tora*; TQD95409.1 is a MYB of *Malus baccata*; Pa, *Prunus avium*; Pp, *P. persica*; Mr, *Morella rubra*; Ci, *Carya illinoinensis*. Purple and blue indicated genes related to polyphenol synthesis and stress response, respectively. Red marked walnut *JrMYB44*. Supplementary Fig. S3 Amino acid sequence alignment and conserved domain of JrMYB44 and AtMYBs. A, Amino acid sequence alignment using blastp of NCBI. JrMYB44 was the Query, while AtMYB44, AtMYB73 and AtMYB70 were Sbjct. B, Conserved domain analysis using Clustal X. Supplementary Fig. S4 The relative expression of *JrMYB44* in transformed lines. A-B, four overexpressed and three suppressed walnut lines transformed by *JrMYB44*. C-D, nine overexpressed and nine suppressed *A. thaliana* lines transformed by *JrMYB44*. E, The relative expression of *AtMYBs* in *JrMYB44* suppression Arabidopsis lines. Supplementary Fig. S5 Phenotype of walnut WT, OE and SE. A, tissue culture seedlings (TCS). B, potted seedlings (PS). Supplementary Fig. S6 Total polyphenol content and components in *JrMYB44* transgenic *A. thaliana*. Aerial parts of WT, OE1, OE2, SE1 during bolting stage were collected for index determination. Error bars represent the SD (n=3). Lowercase indicates significant differences among WT, OE1, OE2, SE1 (*P*<0.05). A, total polyphenol. B, catechin. C, chlorogenic aid. D, syringate. E, p-Coumaric acid. F, quercetin. Supplementary Fig. S7 Drought stress tolerance analysis of *JrMYB44* by homologous overexpression in *J. regia*. WT, OE and SE tissue culture seedlings of the same age were transferred to pot conditions for four months, and treated with no watering for 12 d as drought stress. The significant differences among WT, OE, SE were marked with lowercase (*P*<0.05). A, Leaf phenotype. B-E, water loss, electrolyte leakages (EL) rate, H_2_O_2_ content and MDA content, accordingly. Supplementary Fig. S8 Drought stress tolerance analysis of *JrMYB44* by heterologous overexpression in *A. thaliana*. The significant differences among WT, OE1, OE2, SE1 were marked with lowercase (*P*<0.05). A, NBT staining. B-J, H_2_O_2_ content, MDA content, EL rate, CAT activity, GST activity, SOD activity, total antioxidant capacity, proline content and water loss according to A, accordingly. Supplementary Fig. S9 Expression of genes related to the anthocyanin synthesis pathway in walnut WT, OE and SE lines. The relative expression level was related to the expression of internal reference genes from three repeats. Supplementary Fig. S10 Clustering of DEGs in *A. thaliana* WT, OE1, OE2 and SE1. The relative expression level was related to the expression of internal reference gene. Supplementary Fig. S11 Promoter segments of *JrWRKY7* and *JrDREB2A*. The core sequences of MYB recognition related elements were marked in green or yellow. Segments used in GUS activity assay were underlined. A, *JrWRKY7* promoter. B, *JrDREB2A* promoter. Supplementary Fig. S12 The KEGG pathways based on the transcriptomes of *JrMYB44* overexpression line and WT. A, the flavonoid biosynthesis pathway covering *JrMYB44*, *JrDof1* and *JrWRKY7*. B, the isoflavonoid biosynthesis pathway covering *JrMYB44* and *JrMYC2*, *JrWRKY7*, *JrDREB2A*. Supplementary Fig. S13 Amino acid sequence alignment using blastp of NCBI. A, JrMYC2 (Sbjct) and AtMYC3 (Query). B, JrMYB44 (Sbjct) and AtMYB29 (Query).

## Data Availability

All relevant data are available in the main manuscript and supplementary files.

## Abbreviations

OE: Overexpression
SE: Suppression
TCS: Tissue cultured sterile seedlings
PS: Potted seedlings
TFs: Transcription factors
MBW: MYB-BHLH-WD repeat
DEGs: Differentially expressed genes
GO: Gene ontology
KEGG: Kyoto Encyclopedia of Genes and Genomes
ROS: Reactive oxidative species
CAT: Catalase
GST: Glutathione S-transferase
SOD: Superoxide dismutase
Y1H: Yeast one-hybrid
Y2H: Yeast two-hybrid
DLR: Dual-luciferase reporter assay
LCI: Luciferase complementation imaging assay
GUS: β-Glucuronidase
QDO/X/A: SD-Trp-Leu-His-Ade + X-α-gal + Aureobasidin A
LUC: Luciferase
JA: Jasmonic acid
