# Supplementary material for: JrMYB44 is required for the accumulation of polyphenols and contributes to drought tolerance in Juglans regia
Source: Stress Biol. 2025 Jan 21;5(1):6. doi: 10.1007/s44154-024-00193-7 (PMC11746988; doi:10.1007/s44154-024-00193-7)
Supplement: Supplementary file 1 — Additional file 1: Supplementary Table S1 The primers used for RT-qPCR analysis in walnut. Supplementary Table S2 The primers used for construction of recombinant vectors. Supplementary Table S3 The primers used for RT-qPCR analysis in Arabidopsis. Supplementary Fig. S1 Correlation analysis of drought resistance, JrMYB44 expression and polyphenols. *, **, and *** indicates significant correlation at P<0.05, P<0.01 and P<0.001 level, accordingly. Supplementary Fig. S2 Phylogenetic tree analysis of JrMYB44 protein and its homologs from other species based on sequence alignments of the encoded proteins using neighbor-joining method in MEGA7. Jr, Juglans regia; At, Arabidopsis thaliana; Ga, Gossypium arboretum; Ma, Musa acuminata; Ptr, Populus trichocarpa; Ps, Pisum sativum; Pq, Paeonia qiui; BraA07g032100.3C is a MYB of Brassica rapa, BcaB05g24263 and BcaB03g15272 are MYBs of B. carinata; St, Senna tora; TQD95409.1 is a MYB of Malus baccata; Pa, Prunus avium; Pp, P. persica; Mr, Morella rubra; Ci, Carya illinoinensis. Purple and blue indicated genes related to polyphenol synthesis and stress response, respectively. Red marked walnut JrMYB44. Supplementary Fig. S3 Amino acid sequence alignment and conserved domain of JrMYB44 and AtMYBs. A, Amino acid sequence alignment using blastp of NCBI. JrMYB44 was the Query, while AtMYB44, AtMYB73 and AtMYB70 were Sbjct. B, Conserved domain analysis using Clustal X. Supplementary Fig. S4 The relative expression of JrMYB44 in transformed lines. A-B, four overexpressed and three suppressed walnut lines transformed by JrMYB44. C-D, nine overexpressed and nine suppressed A. thaliana lines transformed by JrMYB44. E, The relative expression of AtMYBs in JrMYB44 suppression Arabidopsis lines. Supplementary Fig. S5 Phenotype of walnut WT, OE and SE. A, tissue culture seedlings (TCS). B, potted seedlings (PS). Supplementary Fig. S6 Total polyphenol content and components in JrMYB44 transgenic A. thaliana. Aerial parts of WT, OE1, OE2, SE1 d [file 44154_2024_193_MOESM1_ESM.pdf]

Supplementary Table S1 The primers for RT-qPCR analysis of the walnut genes

**Gene name**	**5’-primer**	**3’-primer**
18SRNA	5′-GGTCAATCTTCTCGTTCCCTT-3′	5′-TCGCATTTCGCTACGTTCTT-3′
GAPDH	5′-GATCAGCGATGCAATGGAGA-3′	5′-TGCGAACCAATTCCACTGTC-3′
JrMYB44	5’-AGAGACATAGATCGGGTC-3’	5’-ATGATCTGGTCCTCCTCT-3’
JrGSTTau1	5′- GTATGAGTACATAGAAG-3′	5′-TTAATCCAGAACCGAGCT -3′
JrGST2	5′-ATGGCAGCCATCAGACTC-3′	5′-AGTGATTGCTCTTGATTC-3′
JrGST3	5′-ATGGTATTGGAAGATGG-3′	5′-AATTGCAGTAGATTTAAG-3′
JrGST6	5′-ATGAAAGCTGGTGAACAT-3′	5′-TCCATCCACACAGATATG-3′
JrGST7	5′-ATGGTAAGCCAGGAGGTG-3′	5′-ACTCAGCCATCACCTTAC-3′
JrGST8	5′-ATGTCCACGGCTGTGTCCA-3′	5′-GGTTCGTTCCATACAGTC-3′
JrGST9	5′-GAAGAGTGATATCAAGCT-3′	5′-ATGTATTGGACAATGATG-3′
JrGST10	5′-ATGGCATCCGGACTTGTG-3′	5′-TCTCCTTATACCAATCAG-3′
JrGST11	5′-ATGGCGAACAGCAGCAGT-3′	5′-AGAGACTCAGAGATGGAT-3′
JrGST12	5′-ATGGAACAATGAGGTCAG-3′	5′-TGTTGCATAATACCTTAC-3′
JrGSTF8	5′-GCTCATCTCCGTCAACAT-3′	5′-CCACTGTTCTATGGATGC-3′
JrGSTT3	5′-GCGATATGAGCGAACATG-3′	5′-TGGAGACGTCATCTTTCC-3′
JrGSTU8	5′-ACAAGAGTCCTACCCTT-3′	5′-ACAGCATTCTCATGCTG-3′
JrGSTU19	5′-GATCAGGTGATTCTGCTG-3′	5′-GAGGGATTCACATACAGG-3′
JrGSTU23	5’-GTGAAGCTGATTGCCACT-3’	5’-GTCCTTCCATGTCTCCTC-3’
JrAPX1	5’-ATGGCTGCACCAGTAGTT -3’	5’-GTAGAGATCGGCATATGT -3’
JrAPX2	5’- TGCTCACAACTGTCGAAT-3’	5’-CAGACGAAGTACACCAGC-3’
JrAPX3	5’- CGGTACCATGAAGCTAGG-3’	5’-ACAATATCCTGATCACAG -3’
JrGPX1	5’-CAGAACAGCACAGTCCAC-3’	5’-CTAAGATCTGTTCATTG -3’
JrGPX2	5’-ATGGCTACCATGCCGTT-3’	5’- TCCATAGTCGATCTGGCG -3’
JrCAT1	5’-CATGATATTACTCACCTT-3’	5’- AGGCATGGATCACGTCTG -3’
JrSOD1	5’-ATGAGTACTGTGAAAGCT-3’	5’-TAGGAGATCCATGATGCT -3’
JrSOD2	5’-ACAGTAACGATGTCAAG-3’	5’-GATCACCAGCATGACGAT -3’
JrSOD3	5’-ATGCAAGCTGCACTGTCAG-3’	5’- CGTACATTGACAGTTGTG -3’
JrSOD4	5’-ATGGCTGTAGCATTCGCAT -3’	5’- TAGGTTCTCCACATAAGC -3’
JrSOD5	5’- CTAGGTCTAGGCCTTCGC-3’	5’-CAACCTAGGGAACTGTGT -3’
JrSOD6	5’-ACGAGCACTGCCATAGCT-3’	5’-GTACTCCGTCAGTAGCTC -3’
JrPOD1	5’-CATTCTGGCAATTGCAGC-3’	5’-TCATACAGCCTGCTTATG -3’
JrPOD2	5’-ATGGTAGCTCTTGCAGGAG-3’	5’-AGTACATATCTTGGTCTG -3’
JrPOD6	5’-TGTACTATTGGAGATGAT-3’	5’-CATCATCAATAACTTCAT -3’
JrVHAc4	5’-TGTATGGGAGCTGCTTAC-3’	5’-AGAGATGTGCGTAGCCAT-3’
JrWRKY2	5’-CTAGCCAAGCTACACCTG -3’	5’-TCTGCACAATGATTCTAG-3’
JrWRKY7	5’-AGCAGAGTCAGCAGCAAC -3’	5’- GTGCAGAAGCCGAAGGCT -3’
JrWRKY6	5’-GATGAGAACTCGTTGTCT-3’	5’-ATCTGGGAACCTTATTAG-3’
JrWRKY31	5’-AGACGATGTTGATGATTC -3’	5’- ACCAAGCATATCTCTCAG -3’
JrWRKY53	5’-GTACAACATCGTCATCAC-3’	5’-TTGAGATATCGTCAGTGC-3’
JrWRKY70	5’-GACCAGCTAATTGAAGGC-3’	5’-GGAGATACTCCTGCAACT-3’
JrERF02	5’-AGTGGAGCACGAGATCAT-3’	5’-GGATGTTCGAATTGCAGC-3’
JrERF03	5’-ATGGTGAGCTTACGAAGG-3’	5’-AGATGAGCTCGAACCAGT-3’
JrERF15	5’-AGTCAAGCTCCAACTCGT-3’	5’-AGCTCCTCATTCGTACC-3’
JrERF11	5’-ATGTGGCTAGTCCAGCAT-3’	5’-ATTGCACGTCCTCAGCACT-3’

JrERF16	5’-TTCTGGAACTCCGACAG-3’	5’-ATGACTAGGCCATGGAG-3’
JrERF27	5’-TCTGCTCTCACTCAGGTT-3’	5’-TGCATCCTCAGCTGTGT-3’
JrERF47	5’-CTTCGAGCGAAGATGAGT-3’	5’-GGTCTTCTACTGCTCGAT-3’
JrPP2C03	5′-TTGCCATGAACGTCTCCA-3′	5′-AACCAAGGCAGTAGAACC-3′
JrPP2C09	5′-TGGATCTGCTGCTGCTAT-3′	5′-CAGCAGGTTCAAGTATGC-3′
JrPP2C17	5′-TCCACTCCAAGGACAGTT-3′	5′-ACGTATGCATCACCAAGC-3′
JrPP2C28	5′-GGCTATCAATGCAATGGC-3′	5′-TGACATGGTGCTCTGAAG-3′
JrPP2C36	5′-GAAGCGATAACCCTAACC-3′	5′-TCTTCCTCCAGCACTTC-3′
JrDREB1A	5′-GAGAGTTCTCCCTTGTCT-3′	5′-TCCTGGACGTCTTGTTAG-3′
JrDREB1D	5′-AGACTCCGAGAGTAGTAGCT-3′	5′-GCCATATCCTGGACTTCTTG-3′
JrDREB2C	5′-GCATGTCTTCGGAGACAACT-3′	5′-ACGCTGTAGTAACCTACCAG-3′
JrDREB2F	5′-GAAGAGAACTAGACTCTGG-3′	5′-GACATGAACACTAGGTTGG-3′
JrDREB2A	5′-CGTAAGGTTCCAGCCAAGG-3′	5′-AGCCCTTGCAGCTTCATC-3′
JrMYC1	5′-TGAGACCTCACCAACGAT-3′	5′-ATGTCCAAGTCCTGATCG-3′
JrMYC2	5′-CCTGAATGGTGGATCTACTCC-3′	5′-ACCTGACCATCCAATGTTACT-3′
JrDof1	5′-ATGTCCACGCTGTGACTC-3′	5′-GATGCCGTTGACGATGAAG-3′
JrDof2	5′-AGTCACCAACAGATGTCC-3	5′-GAGGCTGACCTCTTGTTT-3
JrDof3	5′-ATGGCTGATCGAGCTCGC-3′	5′-CTATAGGGGGTTTCTGGT-3′
JrDof4	5′-TTCAATGGCTGATCGAGC-3′	5′-AGAGTTGGAACCCGATTG-3′
JrDof6	5′-ACTGAGGATCCTGGCATT-3′	5′-TAGACTCCACCTGCAGTT-3′
JrWD40-1	5′-ATCAAGACGGTTCTGACG-3′	5′-TGAGGAGGCTCTTAAGCT-3′
JrWD40-3	5′-ATCAACGGTGACCTTCAC-3′	5′-CTTCATCATCACCCGATG-3′
JrWD40-15	5′-TACAAGCTCCACTACGAG-3′	5′-TCTTACTGCCACCATTCC-3′
JrWD40-18	5′-TGAGGAGCCTGATGATTC-3′	5′-TAAGTCCACCCAAGCATC-3′
JrWD40-26	5′-TTCTCTGGATGATGCAGC-3′	5′-ATGATCCTTCAGGACCTG-3′
JrWD40-35	5′-AAGTGGAGATGGTGACAG-3′	5′-TCAGAGTCCTCATCATCC-3′

Supplementary Table S2 The primers used for gene clone and construction of recombinant vectors.

**Gene/Construct**	**5'-primer (5'—3')**	**3'-primer (5'—3')**
35S::JrMYB44	ATCGTCTAGAATGGAGGCGTCACAGAGAGAC	CGATGGTACCTCAAAGTTCAAGCCTGGCCAT
35S::JrWRKY7	ATCGGGATCCATGGCCGTGGAGTTCATGATG	CGATGGTACCTCAAGAGGATTCTAGGATCAG
35S::JrDREB2A	ATCGTCTAGAATGGCTGACGGGCAAAGAAG	CGATGGTACCTCATAAAAGCATTTCCAAGT
35S::JrMYC2	ATCGGGATCCATGGACGAGATTATCTCTTCC	CGATGGTACCCTAACTCCGCATACTTTGCAG
JrDof1	TCGGGATCCATGCAAGACCCAGCAACATTC	CGATGGTACCCTACTGAGATCTTGAACCTGG
pHis2-Motif1	AATTCAACGGAACGGAACGGGAGCT	CCCGTTCCGTTCCGTTG
pHis2-Motif1M	AATTCGGACCGGACCGGACCGAGCT	CGTCCGGTCCGGTCCG
pHis2-Motif1S	GGAATTCTGTGAACGGTAATCGATG	CTCTCAGAACCATCAAAGGAGCTCG
pHis2-Motif1SM1	GGAATTCTGTG GGACC TAATCGATG	
pHis2-Motif1SM2	GGAATTCTGTGTAATCGATGAAAAT	
pCAM-Motif1	GATCCAACGGAACGGAACGGCCCTTCCTCTATATAAGGAAGTTCAT<br>TTCATTTGGAGAGAACACGGA	AGCTTCCGTGTTCTCTCCAAATGAAATGAACTTCCTTATATAGAGGAA<br>GGGCCGTTCCGTTCCGTTG
pCAM-Motif1M	GATCCGGACCGGACCGGACCCCCTTCCTCTATATAAGGAAGTTCATT<br>TCATTTGGAGAGAACACGGA	AGCTTCCGTGTTCTCTCCAAATGAAATGAACTTCCTTATATAGAGGAA<br>GGGGGTCCGGTCCGGTCCG
pCAM-Motif1S	GGATCCTGTGAACGGTAATCGATG	AAGCTTCCGTGTTCTCTCCAAATGAAATGAACTTCCTTATATAGAGGA<br>AGGGCTCTCAGAACCATCAAAG
pCAM-Motif1SM1	GGATCCTGTG GGACC TAATCGATG	
pCAM-Motif1SM2	GGATCCTGTGTAATCGATGAAAAT	
pHis2-Motif2	AATTCCGGTTGCGGTTGCGGTTGGAGCT	CCAACCGCAACCGCAACCGG
pHis2-Motif2M	AATTCATTCCTATTCCTATTCCTGAGCT	CAGGAATAGGAATAGGAATG
pHis2-Motif2S	GGAATTCGATCGGTTGACAGAGTTAC	CTCTCAGACTGGGGACTCGGAGCTCG
pHis2-Motif2SM1	GGAATTCGAT ATTCCTACAGAGTTAC	
pHis2-Motif2SM2	GGAATTCGATACAGAGTTACTTCGTC	
pCAM-Motif2	GATCCCGGTTGCGGTTGCGGTTGCCCTTCCTCTATATAAGGAAGTTC<br>ATTTCATTTGGAGAGAACACGGA	AGCTTCCGTGTTCTCTCCAAATGAAATGAACTTCCTTATATAGAGGAA<br>GGGCAACCGCAACCGCAACCGG
pCAM-Motif2M	GATCCATTCCTATTCCTATTCCTCCCTTCCTCTATATAAGGAAGTTCA<br>TTTCATTTGGAGAGAACACGGA	AGCTTCCGTGTTCTCTCCAAATGAAATGAACTTCCTTATATAGAGGAA<br>GGGAGGAATAGGAATAGGAATG
pCAM-Motif2S	GGATCCGATCGGTTGACAGAGTTAC	AAGCTTCCGTGTTCTCTCCAAATGAAATGAACTTCCTTATATAGAGGA<br>AGGGCTCTCAGACTGGGGACTCG
pCAM-Motif2SM1	GGATCCGATATTCCTACAGAGTTAC	

pCAM-Motif2SM2	GGATCCGATACAGAGTTACTTCGTC	
pHis2	GCCTTCGTTTATCTTGCCTGCTC	CGATCGGTGCGGGCCTCTTC
pCAMBIA1301	TAGAGTCGACCTGCAGGCAT	ATCATCATCATAGACACACG
pROKII	TTTCATTTGGAGAGAACACG	TGCCAAATGTTTGAACGATC
pGAD	CTATTCGATGATGAAGATACCCCACCAAACCC-3'	GTGAACTTGCGGGGTTTTTCAGTATCTACG-3'
pGAD-JrMYB44	TGGCCATTATGGCCCGGGATGGAGGCGTCACAGAGAG	GACATGTTTTTCCCGGGTCAAAGTTCAAGCCTGGCCAT
AD-JrWRKY2	TGGCCATTATGGCCCGGGATGTCAAAGAAGGACCAGGAAT	GACATGTTTTTCCCGGGCTATGTTATCTGCTCTTCTT
BD-JrWRKY7	CATGGAGGCCGAATTCATGGCCGTGGAGTTCATGATG	GCAGGTCGACGGATCCTCAAGAGGATTCTAGGATCAG
pSupre1300-JrMYB44	ATCGTCTAGAATGGAGGCGTCACAGAGAGAC	CGATGGTACCTCAAAGTTCAAGCCTGGCCAT
pGreenI-JrWRKY7	CGATGGTACCATGGCCGTGGAGTTCATGAT	ATCGGGATCCTCAAGAGGATTCTAGGATCAG
pGreenII-JrDREB2A	CGATGGTACCATGGCTGACGGGCAAAGAAG	ATCGGGATCCTCATAAAAGCATTTCCAAG
AD-JrMYB44	TGGCCATTATGGCCCGGGATGGAGGCGTCACAGAGAGAC'	GACATGTTTTTCCCGGG TCAAAGTTCAAGCCTGGCCAT
BD-JrMYB44	CATGGAGGCCGAATTC ATGGAGGCGTCACAGAGAGAC	AAGGAAAAAAGCGGCCGCTCAAAGTTCAAGCCTGGCCAT
AD-JrMYC2	TGGCCATTATGGCCCGGGATGGACGAGATTATCTCTTCC	GACATGTTTTTCCCGGGCTAACTCCGCATACTTTGCAG
BD-JrMYC2	CATGGAGGCCGAATTCATGGACGAGATTATCTCTTCC	GCAGGTCGACGGATCCCTAACTCCGCATACTTTGCAG
AD-JrDof1	CTAGTCTCTAGAGGATCCATGCAAGACCCAGCAACATT	AGGTCGACGGATCCCTACTGAGATCTTGAACCTGG
BD-JrDof1	GCAGGTCGACGGATCCATGCAAGACCCAGCAACATTC	AAGGAAAAAAGCGGCCGCCTACTGAGATCTTGAACCTGG
pFGC-JrMYB44-Cis	ATCGTCTAGAGTCAGGCAAGTCGTGCCG	CGATGGATCCGGTGTCATCGCTCATCAT
pFGC-JrMYB44-Anti	ATCGGGTACCGGTGTCATCGCTCATCAT	CGATGAGCTCGTCAGGCAAGTCGTGCCG
NLuc-JrMYB44	CGATGGTACCATGGAGGCGTCACAGAGAGAC	ATCGTCTAGATCAAAGTTCAAGCCTGGCCAT
CLuc-JrMYC2	CGATGGTACCATGGACGAGATTATCTCTTCC	ATCGGGATCCCTAACTCCGCATACTTTGCAG
CLuc-JrDof1	CGATGGTACCATGCAAGACCCAGCAACATTC	ATCGGGATCCCTACTGAGATCTTGAACCTGG
JrMYB44-His	TAAGAAGGAGATATACAT ATGGAGGCGTCACAGAGAGACAT	GTGGTGGTGGTGGTGCTCGAGTAAGTTCAAGCCTGGCCATGTAAC
GST-JrDof1	GATCTGGATCCGCGTGGATCCATGCAAGACCCAGCAACATT	GTCACGATGGTCGACCTCGACTACTGAGATCTTGAACCTGG

Supplementary Table S3 Primers for RT-qPCR of the *Arabidopsis thaliana*.

Gene name	Accession No.	5'-primer	3'-primer
AtSnRK2.2	AT3G50500.2	5'-CGCCTTCCCATTTGGCTAT-3'	5'-AGGTGCCGGACTTCCATCT-3'
AtSnRK2.3	AT5G66880.1	5'-ATGCACGACAGTGATCGA-3'	5'-TGATAGCCAGATGAGTCG-3'
AtSnRK2.6	AT4G33950.1	5'-GGTATGAACTCGTCAAGG-3'	5'-GTTCTCCTCCAGATGCAT-3'
AtPYL1	AT5G46790.1	5'-GTTGCTCATCTCTCCTAGC-3'	5'-TCTCCGATCATCGTCCAAC-3'
AtPYL2	AT2G26040.1	5'-TCATAACCCAACGCATCC-3'	5'-TCGTCATCGACGAACTCA-3'
AtPYL3	AT1G73000.1	5'-CAGAGTTAACGGTAACGG-3'	5'-GAACTCGTTGACCGATGT-3'
AtPYL4	AT2G38310.1	5'-ATGATGATCGCGTCGTTTC-3'	5'-GGAGGCTACCAACGTTATC-3'
AtPYL5	AT5G05440.1	5'-GGTCCGATCAAGAGAGTGT-3'	5'-TACGGCACTGTCTGATGAAG-3'
AtABF1	AT1G49720.2	5'-TGATGAGCTTCAGAGCAC-3'	5'-CAGACTTCATCGACAGTC-3'
AtABF2	AT1G45249.3	5'-TCGATATACTCGTTGACG-3'	5'-TCTGACTAAGCGTTCGAG-3'
AtABF3	AT4G34000.1	5'-GTCTATGAACATGGATGA-3'	5'-AGTGAACCTTGTCTCTGC-3'
AtABF4	AT3G19290.3	5'-ATGGACTGCTGAGGAAGC-3'	5'-GAGGCACATTCGACTCAC-3'
AtDREB1A	NM_118680.1	5'-TCGTGAGACTCGTCACC-3'	5'-GATTCGGAGTCTCCAAGC-3'
AtDREB1B	AB013816.1	5'-TCGTGAGACTCGTCACC-3'	5'-AAGTTGAGACATGCTGAT-3'
AtDREB1C	NM_118679.1	5'-GCTGAGATGGCAGCTCGT-3'	5'-ATGAGCATCCGTCGTCAT-3'
AtDREB2A	NM_120623.2	5'-TACTACTGTGGCTGAGAG-3'	5'-CTGCTACCTCGATTAGG-3'
AtDREB2B	NM_111939.2	5'-AGAGTACAACGAGATTGT-3'	5'-ACCGTACATAGCGGTAGC-3'
AtDREB2C	NM_129594.2	5'-TGGAGAGAGTACAATGAG-3'	5'-GCTTCATATGAACTGGAG-3'
AtDREB2G	NM_121850.1	5'-ACTCTATGGACACGAGGC-3'	5'-CACTTGAAGACGAACATC-3'
AtSOD1	AT1G08830.1	5'-ATGGCGAAAGGAGTTGCAG-3'	5'-TGTCGATTAGCATCCTCAG-3'
AtSOD2	AT2G28190.1	5'-ATGGCTGCCACCAACAC-3'	5'-CTGAGTCATCTTGGGTC-3'
AtSOD3	AT1G12520.1	5'-GTCAGTGGCAACGACTTCAG-3'	5'-GCTGAGGCATGGCTCGATC-3'
AtP5CS1	AT2G39800.1	5'-ACGTATCGTCGTTAAGGT-3'	5'-TGAGGCTTCTGAAGATCC-3'
AtP5CS2	AT3G55610.1	5'-AGGTGGAAGATTGGCTCT-3'	5'-GTAAGCCATGAGACTGCTC-3'
AtGSTT1	AT5G41210.1	5'-CAGATCGAATGTCACAG-3'	5'-TGATCAGCAACACTTGG-3'
AtGSTT2	AT5G41240.1	5'-GTTGATGGCAGACTTAAGC-3'	5'-TTCTCAGCTTCAGCAGCT-3'
AtGSTT3	AT5G41220.1	5'-GTGTATGCGGATCGAATG-3'	5'-CTACACTTGGGTATGCTG-3'
AtGSTU1	AT2G29490.1	5'-GCGTGCCATACGAATACT-3'	5'-GGATCTGGTCATCGATG-3'
AtGSTU2	AT2G29480.1	5'-AGTCGTAGAGTCGAGATG-3'	5'-TAGGGATCGTGAGGTAG-3'
AtGSTU3	AT2G29470.1	5'-TTCAGTCGTAGAGTCGAG-3'	5'-CATATGGACTTTGCGGTAG-3'
AtGSTU4	AT2G29460.1	5'-GTGCCTTACGAGTACTTG-3'	5'-TGGTCCAACCTGTTCATC-3'
AtGSTU5	AT2G29450.1	5'-CATACCGTACGAGTACGT-3'	5'-AATCTGTTCATCGACGAG-3'
AtGSTU6	AT2G29440.1	5'-GGTGTACCGTACGAATAC-3'	5'-AAGACTCGAGCCTTGGAT-3'
AtGSTU7	AT2G29420.1	5'-ATGAGTTCCTGGAGCAAG-3'	5'-AGACCAGAATCGAGCCAT-3'
AtGSTU8	AT3G09270.1	5'-GAGTAGAGATGGTCCT-3'	5'-GCACGTTCATAAGGATC-3'
AtGSTU9	AT5G62480.1	5'-ACTCCATGGATCATTCGC-3'	5'-AAGGACTCAGAGATGGGT-3'
AtGSTU10	AT1G74590.1	5'-ACATGGGACGTGGATAAG-3'	5'-TGTCCACGTCTCATCGAT-3'
AtGSTU11	AT1G69930.1	5'-AGCTACTAGGAGCATGGC-3'	5'-GAGCCAAGTCTCATCAAC-3'
AtGSTU12	AT1G69920.1	5'-CGAATACTACTGTGAAGC-3'	5'-GACGTATTGGACGATGTTG-3'
AtGSTU13	AT1G27130.1	5'-CTGTCAAGTACGAGTAC-3'	5'-GTCATCGATGTACTGAG-3'
AtGSTU14	AT1G27140.1	5'-ACCTGATGATGATCTTGGG-3'	5'-GAAGCACTTGTCGTCGAT-3'
AtGSTU15	AT1G59670.1	5'-CGGATCTAAGAGTGAGCT-3'	5'-CGGTAACCACTTGTCATC-3'
AtGSTU16	AT1G59700.1	5'-AGGAGTATGGTACAGTCC-3'	5'-GTTCCACGTCTCATCAATG-3'
AtGSTU17	AT1G10370.1	5'-GAAGCTGATCGGTGCATG-3'	5'-TGGACTCACTCACCGGTTT-3'

AtGSTU18	AT1G10360.1	5'-TGAAGCTGATCGGCTCAT-3'	5'-AGTTCCAAGCCTCGTCGAT-3'
AtGSTU19	AT1G78380.1	5'-ATGAGGACAAGGATCGCA-3'	5'-AGAACCTAGCTTGAGCTC-3'
AtGSTU20	AT1G78370.1	5'-AGTATGTTCGGGATGAGG-3'	5'-GACGTACTGGACAACGTT-3'
AtAPX1	AT1G07890.1	5'-CCGTGAGCGAAGATTACAAG-3'	5'-GAGCAATGTGGATACCACTG-3'
AtAPX2	AT3G09640.1	5'-GCTGTTCAGAGATGCAAG-3'	5'-GGAACAGCTCCTTGATAG-3'
AtWRKY71	AT1G29860.1	5'-CTCACTAGAGAAGGTCTTC-3'	5'-TCACCAACACCATGTAGGT-3'
AtWRKY63	AT1G66600.1	5'-ACATCGATCACAAGGCTG-3'	5'-TTTGAGGCACAAGACCTG-3'
AtWRKY57	AT1G69310.1	5'-ATCTGAGCAACGACGACT-3'	5'-GAACTTGAGGTAGCTGAG-3'
ATWRKY1	AT2G04880.1	5'-CATACCAGTGGCTGTAAC-3'	5'-CAGTTGTATCCGTCTTCC-3'
ATWRKY25	AT2G30250.1	5'-ACTATCCAGAGAGAACCG-3'	5'-GGTTGAGATTGTAGCTGC-3'
AtPP2C3	AT2G29380.1	5'-AAGTAGTGACCGACGCATGT-3'	5'-TGGACTGGATACGTCGACAT-3'
AtPP2CF1	AT3G05640.1	5'-GCTAGATCCTTCTCGATC-3'	5'-GATGGCACAGTCCTGATT-3'
AtDOF1	AT1G51700.1	5'-CAGTCGATGATGATGACG-3'	5'-ACTAGACGGAGAAGAACC-3'
AtDOF2.1	AT2G28510.1	5'-GTCCAAGATGTGAATCTCC-3'	5'-GGTTCTGAAGTGGATCAGT-3'
AtDOF2.4	AT2G37590.1	5'-TGGAGGTCATGTTCTTCG-3'	5'-TGAGTGAGGCTGTAGTTG-3'
AtDOF2	AT3G21270.1	5'-TGATGGCGAAGCAACAAC-3'	5'-TGGTTGCGTTCTTACGAG-3'
AtWD401	AT1G24130.1	5'-TCCTCAACGTCATCATCG-3'	5'-TTGGCAACTACGTTACGG-3'
AtWD402	AT1G24530.1	5'-TCATCTTCCTCACTCAGC-3'	5'-CTTACTTCGTGTCCAGAC-3'
AtWD403	AT1G29320.2	5'-AAGTGACTGAAGCTCGTG-3'	5'-TCTGAGACTCACATCTCC-3'
Actin2	AT3G18780	5'-TGCAGGAGATGATGCTCC-3'	5'-ATACGAAGCTCATTGTAG-3'
GAPDH	AT1G13440	5′-TCTCGATCTCAATTTCGCAAAA-3′	5′-CGAAACCGTTGATTCCGATTC-3′
AtMYB44	AT5G67300.1	5′-CATGGAGTCCTGAAGAAG-3′	5′-AGAAGACGAGCAATCGTC-3′
AtMYB70	AT2G23290.1	5′-AAGGACCATGGAGTCCT-3′	5′-AGTGCGACCATTGAGAAG-3′
AtMYB73	AT4G37260.1	5′-ATGATCTGTTGCAGAGGC-3′	5′-GGTTCGTCCATTGAGAAG-3′

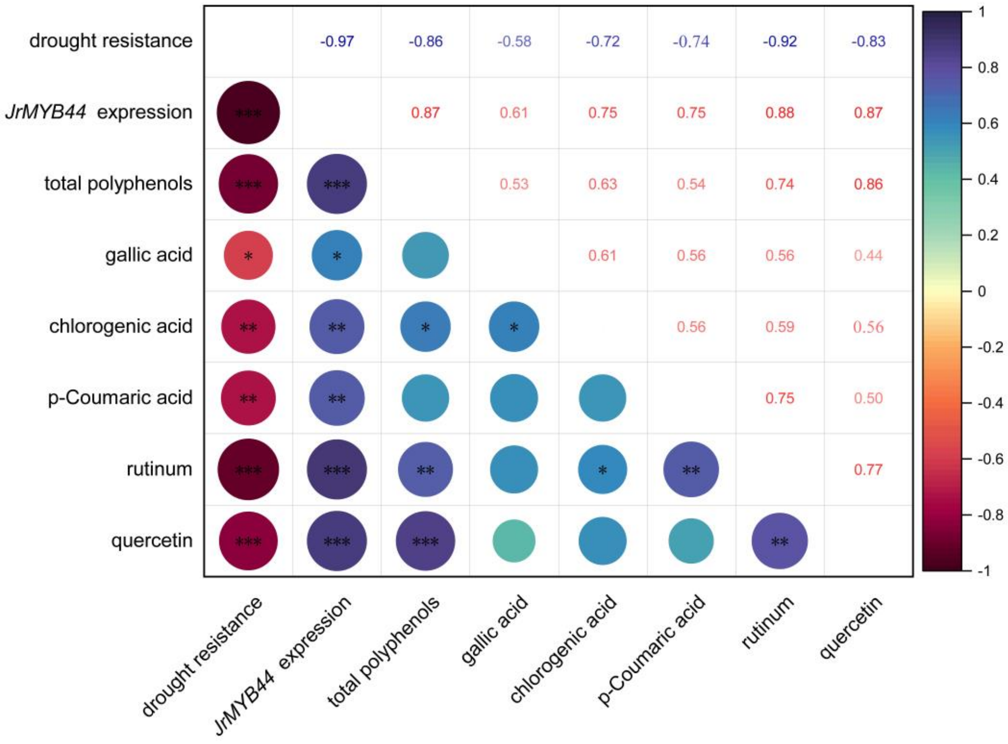

Supplementary Fig. S1 Correlation analysis of drought resistance, *JrMYB44* expression and polyphenols. *, **, and *** indicates significant correlation at *P*<0.05, *P*<0.01 and *P*<0.001 level, accordingly.

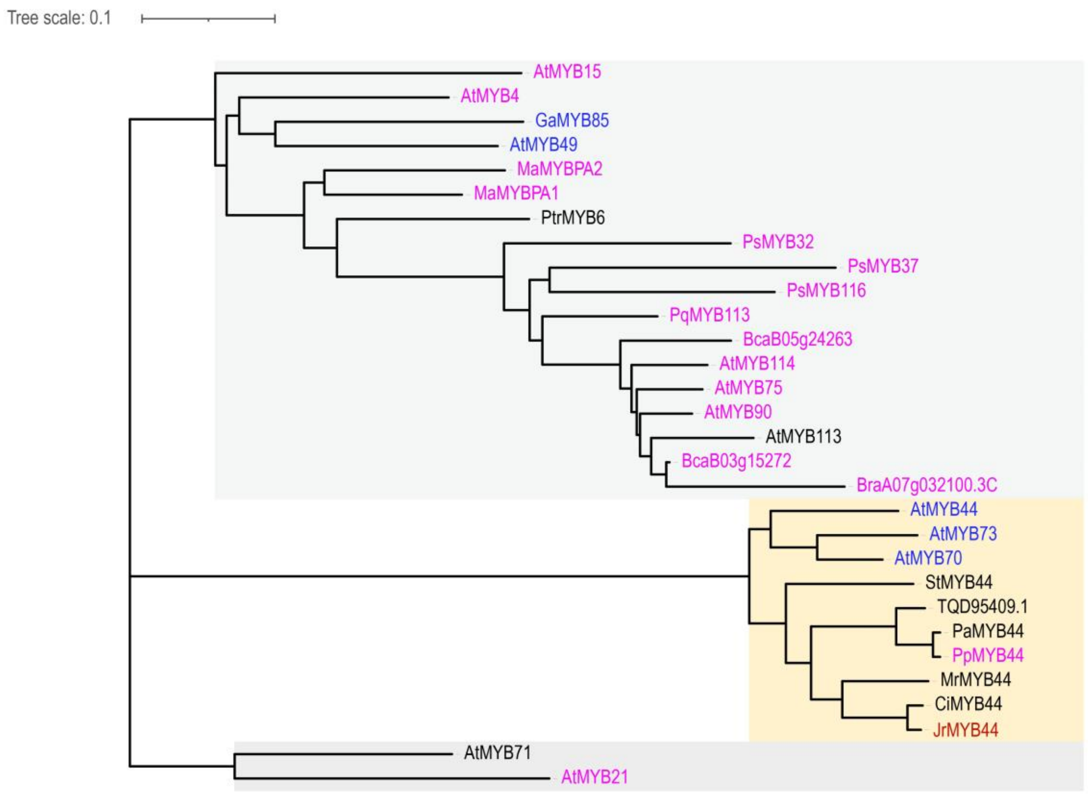

Supplementary Fig. S2 Phylogenetic tree analysis of JrMYB44 protein and its homologs from other species based on sequence alignments of the encoded proteins using neighbor-joining method in MEGA7. Jr, *Juglans regia*; At, *Arabidopsis thaliana*; Ga, *Gossypium arboretum*; Ma, *Musa acuminata*; Ptr, *Populus trichocarpa*; Ps, *Pisum sativum*; Pq, *Paeonia qiui*; BraA07g032100.3C is a MYB of *Brassica rapa*, BcaB05g24263 and BcaB03g15272 are MYBs of *B. carinata*; St, *Senna tora*; TQD95409.1 is a MYB of *Malus baccata*; Pa, *Prunus avium*; Pp, *P. persica*; Mr, *Morella rubra*; Ci, *Carya illinoinensis*. Purple and blue indicated genes related to polyphenol synthesis and stress response, respectively. Red marked walnut JrMYB44.

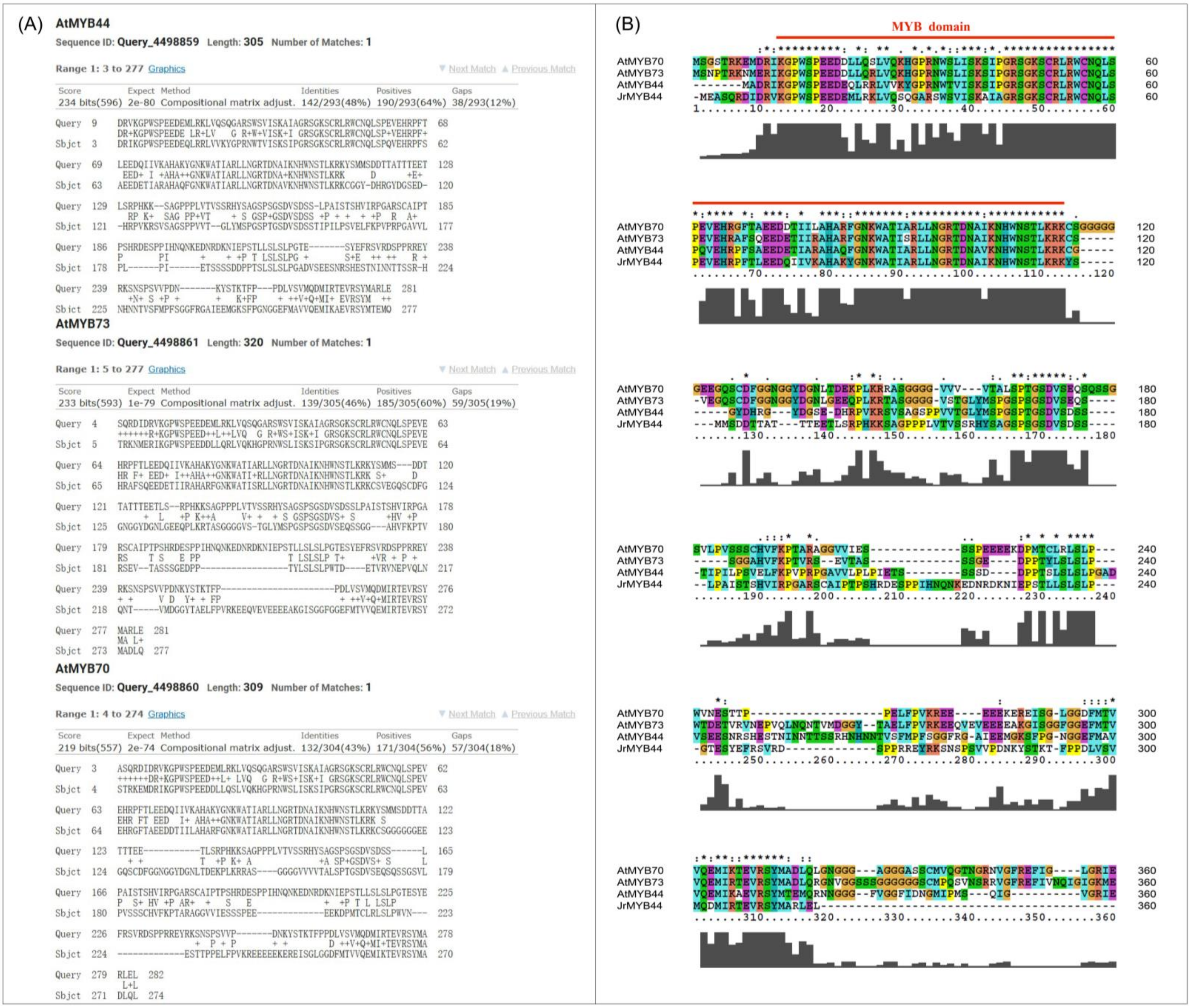

Supplementary Fig. S3 Amino acid sequence alignment and conserved domain of JrMYB44 and AtMYBs. A, Amino acid sequence alignment using blastp of NCBI. JrMYB44 was the Query, while AtMYB44, AtMYB73 and AtMYB70 were Sbjct. B, Conserved domain analysis using Clustal X.

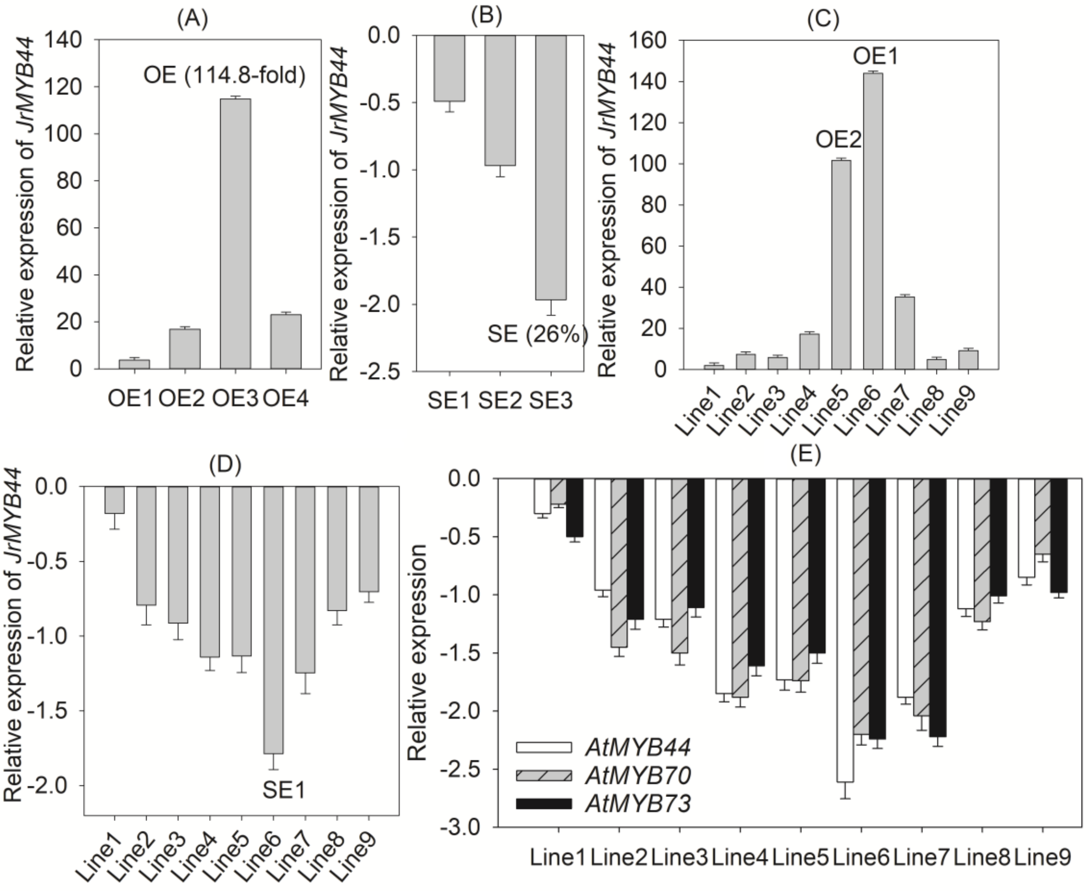

Supplementary Fig. S4 The relative expression of *JrMYB44* in transformed lines. A-B, four overexpressed and three suppressed walnut lines transformed by *JrMYB44*. C-D, nine overexpressed and nine suppressed *A. thaliana* lines transformed by *JrMYB44*. E, The relative expression of *AtMYBs* in *JrMYB44* suppression *Arabidopsis* lines.

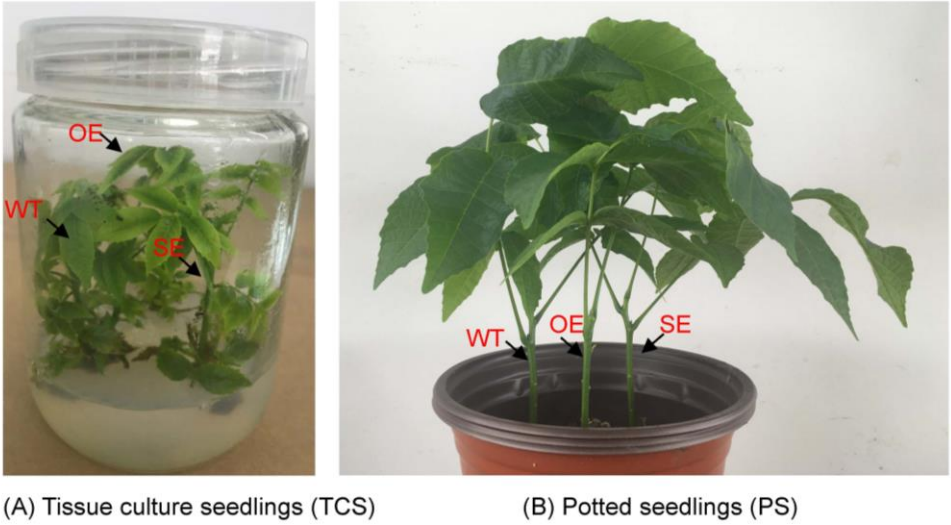

Supplementary Fig. S5 Phenotype of walnut WT, OE and SE. A, tissue culture seedlings (TCS). B, potted seedlings (PS).

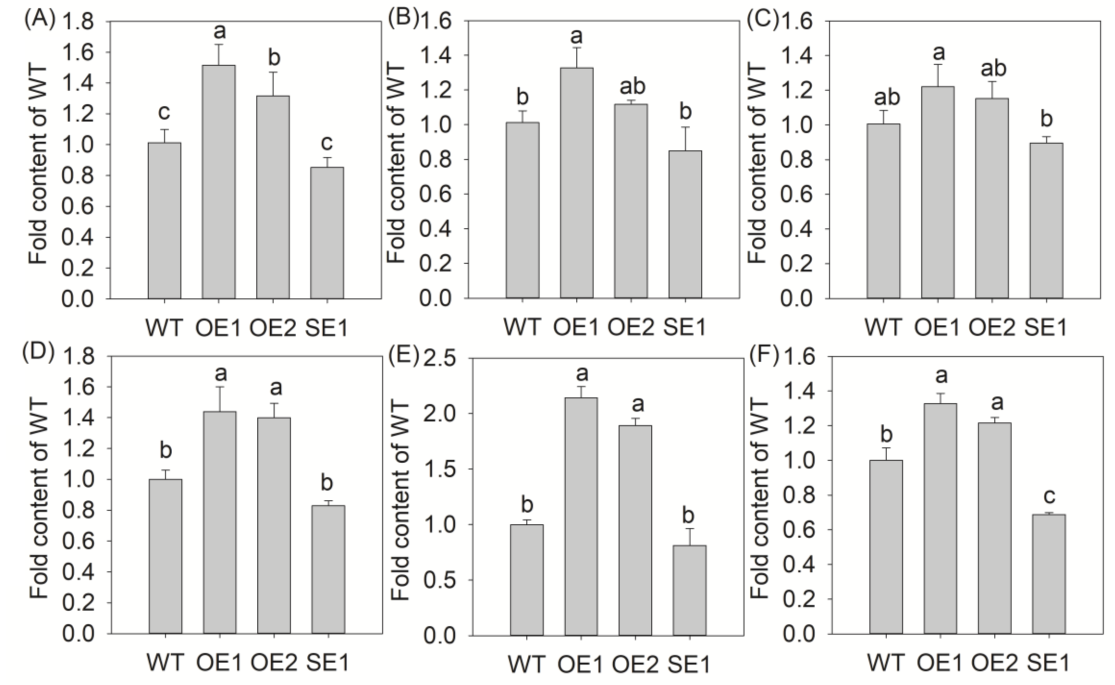

Supplementary Fig. S6 Total polyphenol content and components in *JrMYB44* transgenic *A. thaliana*. Aerial parts of WT, OE1, OE2, SE1 during bolting stage were collected for index determination. Error bars represent the SD (n=3). Lowercase indicates significant differences among WT, OE1, OE2, SE1 (P<0.05). A, total polyphenol. B, catechin. C, chlorogenic aid. D, syringate. E, p-Coumaric acid. F, quercetin.

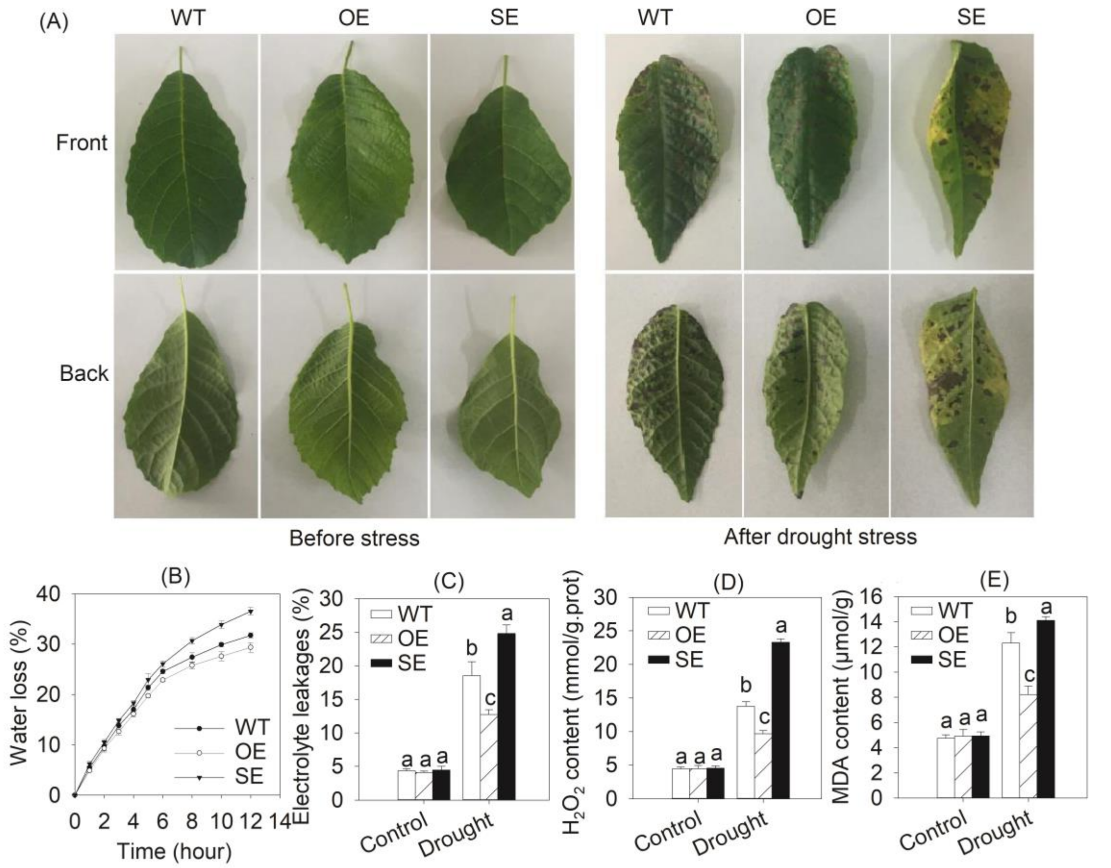

Supplementary Fig. S7 Drought stress tolerance analysis of *JrMYB44* by homologous transient overexpression in *J. regia*. WT, OE and SE tissue culture seedlings of the same age were transferred to pot conditions for four months, and treated with no watering for 12 d as drought stress. The significant differences among WT, OE, SE were marked with lowercase (P<0.05). A, Leaf phenotype. B-E, water loss, electrolyte leakages (EL) rate, H_2O_2 content and MDA content, accordingly.

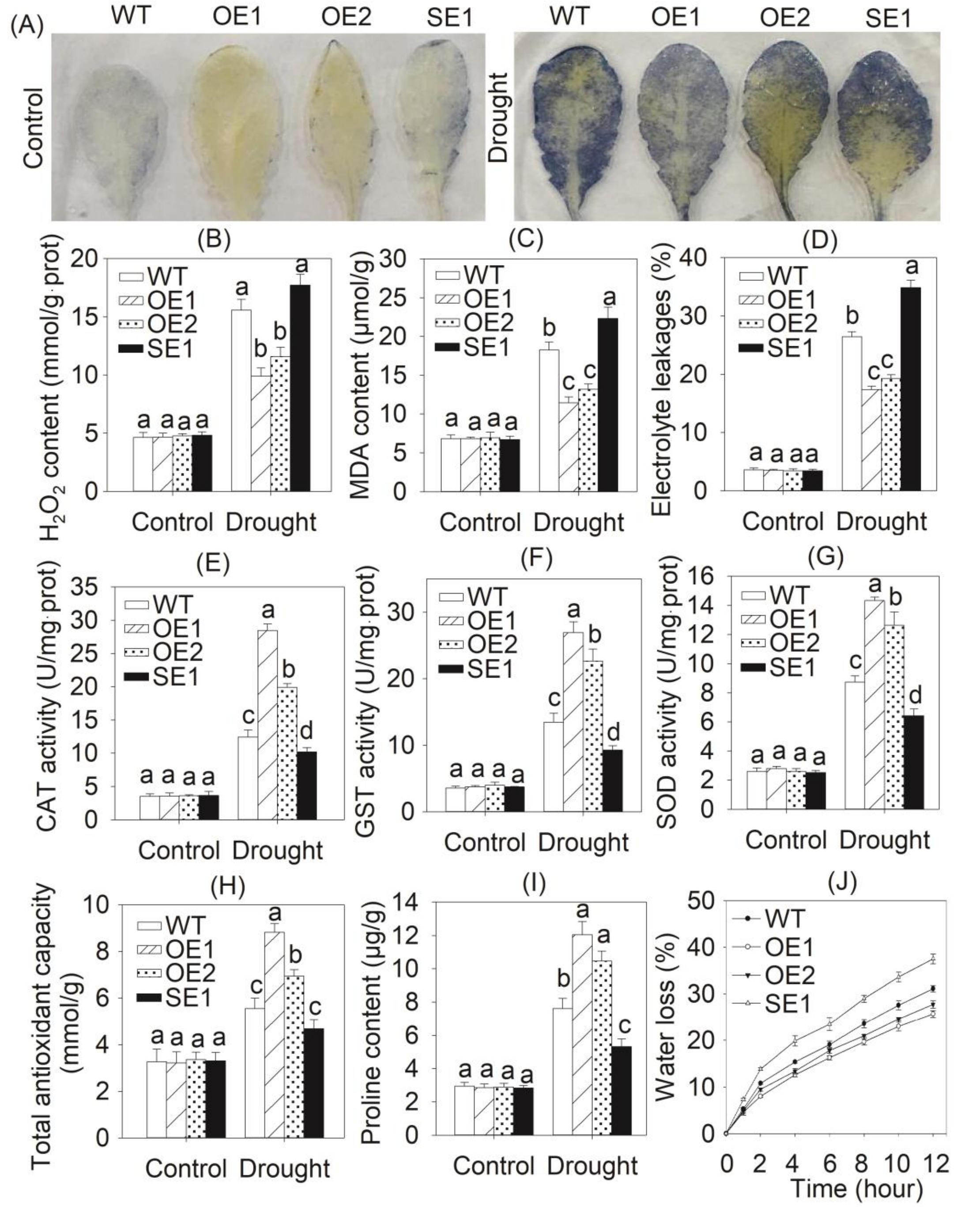

Supplementary Fig. S8 Drought stress tolerance analysis of *JrMYB44* by heterologous overexpression in *A. thaliana*. The significant differences among WT, OE, SE were marked with lowercase ($P<0.05$). A, NBT staining. B-J, H_2O_2 content, MDA content, electrolyte leakages (EL) rate, CAT activity, GST activity, SOD activity, total antioxidant capacity, proline content and water loss according to A, accordingly.

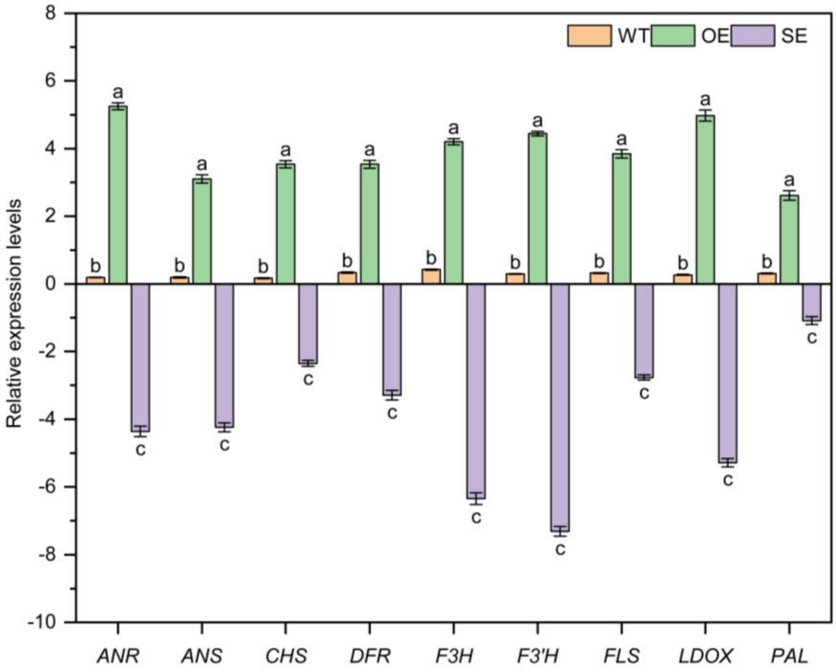

Supplementary Fig. S9 Expression of genes related to the anthocyanin synthesis pathway in walnut WT, OE and SE lines. The relative expression level was related to the expression of internal reference genes from three repeats.

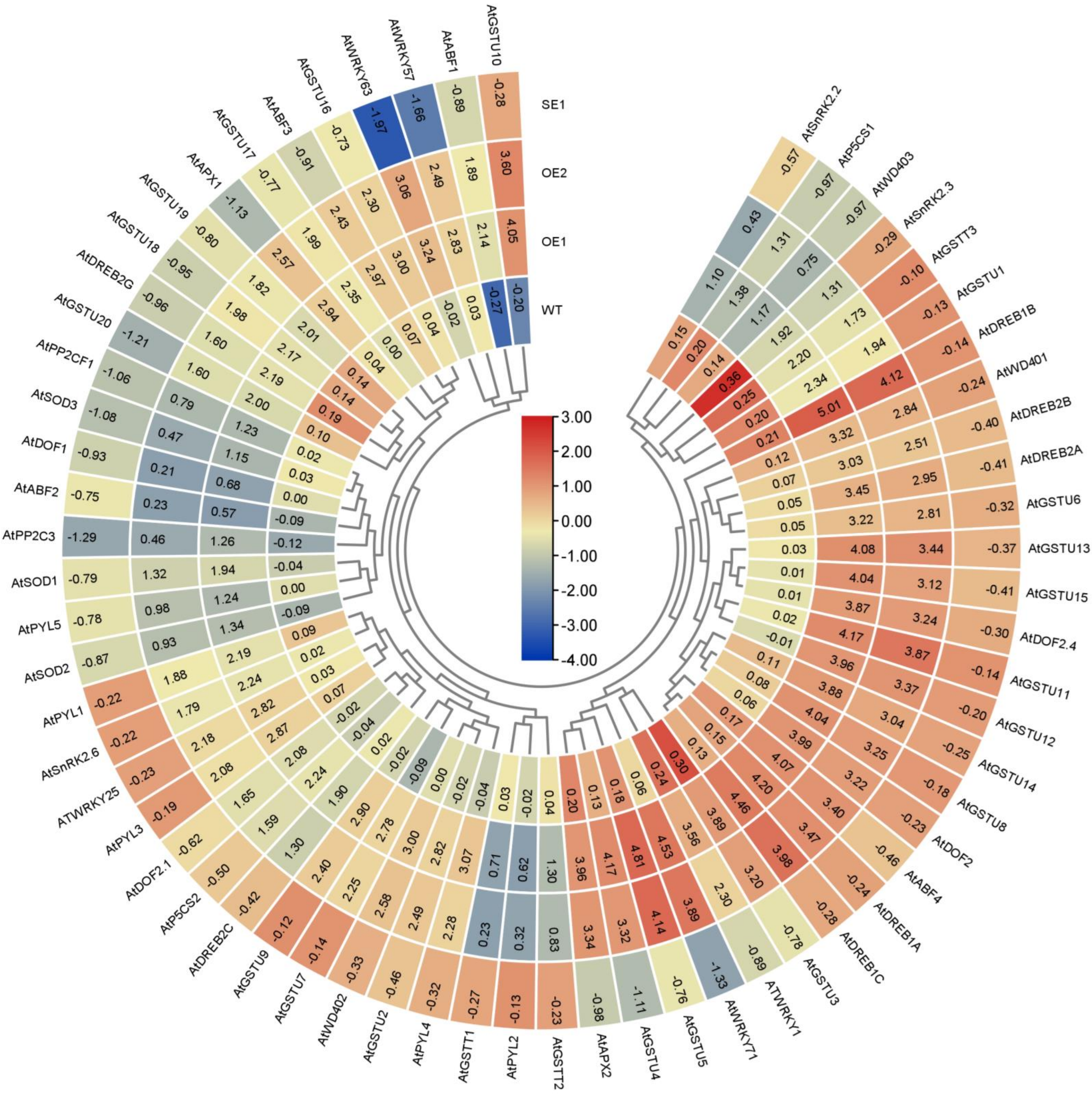

Supplementary Fig. S10 Clustering of DEGs in *A. thaliana* WT, OE1, OE2 and SE1.

The relative expression level was related to the expression of internal reference gene.

(A)

>*JrWRKY7* Promoter 1038 bp

GTGCGTATGATATATCTTTGTCAAAGGAAATCAAATCCAGTTTAGTCAAGCATAGGAAGGG
ACCTCAATTTGTGGGTGGGCACAATAAAATAAGTTTAGTACAAAATAGCAT*TGAC*TATTCTA
CGACTAGTCTATATTTAAAATTTAAAAAAAAATAATATTTACAATTTTAGATTATGCAAGTAAGTA
TGAGAGATTTACATGTAAAAAATTAATTTTTTAATAATAAATTCTATTATTTTTTAAAAAGAATA
AGTAGAGTTTGTACGTCATAAAACTGTATCTAGCAGTACTCTTAATCTACATTTAATTTAATC
TGATTGGTTAAAAACTAGCGATAATTACAGAAAATGTGTAACAAATACAATCAAGTTAATTGA
GAATTAGGCATAGGTTTGTTGATATTGCAGAGAAAAACAGACCATACATGTAACGAATCAAA
TATGTGGTAGAAAAGGAAATGAACATAGGGAGATTTGAAAGTACCGGAAAAACAACAACAA
AACAATAAAGACATCCTTATTATTAACAACTCCAATCAAAGTACTGTTTCAAAGCCCAGCAT
TACGAAGTCAACTCTACCGTGCTGGCAGGCAACAACAAATTAACCCACTTTCCTCCTCCA
ACACAGATCGAATGGTTAATTGCCAATGGTT*TGAC*CTGATGTACGCCAATCTCAATTCACA
ACGTTCGTAAAGACTACATTAACCTTAACAGGCCCCACATCGATCAGATGAGGTCAATGTC
G*TGAC*TAATAAAAAAAAAAAGGTTAAAAAATAGGAAATAAAATTAAAAATTAATTTTGTGAACG
GTAATCGATGAAAATTGTCATGTAGTTGGGGGCCTGATTCGCACCTACGGTT*TGAC*GGGG
GCCGCTAGCG*TGAC*AGGTCGGTCACGCTGTGGGCCAGTAGGGGACTGAGGAGGGCTAA
AAATTTAATGGGCACCTCTT*TGAC*CAGAGACCCACCTTTCGTTTTATTAGCTCTCCTCGTT
CCTTTTTACCCTTTTCCTCTTTCGTTCTCTGTCTTTGATGGTTCTGAGAG

AMYBOX1	355 (+) TAACARA	S000020
MYBGAHV	355 (+) TAACAAA	S000181
MYBCOREATCYCB1	806 (+) AACGG	S000502

The underlined part was used in Y1H and GUS activity assay (Motif1S)

(B)

>*JrDREB2A* Promoter 506 bp

AAACCACTTTACACACACGCGTCATGTCCACCTTAAAATGGAAATGAAACCAAGAATTAAA
CTTACCTTATACAGAAGAGAGCTTTGGATGCCGAGATAAATAGCCACAAGCCACGTGTCTC
AAATCTATCCGAGGATATGACACGGCAAGATAAATACGATAGCCACGTGATCGCATTACATA
AATATAGCCTTTATAGATAGATATGCATTCGCGGAAACATCGCAGCAACAGAGAGAGTGCC
TGATGGCATGTTGTTATTCTCTCTCTCTCTCTCTCTCTCTCTCTCTCTCTCTATATATATAT
ATATATATATATCGATATCCTTTGTTGATCGGTTGACAGAGTTACTTCGTCTCCAAGGCAAA
GGTCGCAATACCAGCGTTATCAAGACAAACAGAGCAAGCACTGCAAAACGCTACAACAAA
GAAACAAATATCATATATAGAGCTTAGAACTCGAGCTTCAACCGAGGCTTTTTGAACGAGT
CCCCAGTCTGAGAG

MYB1AT	1 (+) WAACCA	S000408
MYB1AT	47(+) WAACCA	S000408
MYBST1	135 (+) GGATA	S000180
MYBCORE	339 (+) CNGTTR	S000176

The underlined part was used in Y1H and GUS activity assay (Motif2S)

Supplementary Fig. S11 Promoter segments of *JrWRKY7* and *JrDREB2A*. The core sequences of *MYB* recognition related *cis*-elements were marked in green or yellow. Segments used in GUS activity assay were underlined. A, *JrWRKY7* promoter. B, *JrDREB2A* promoter.

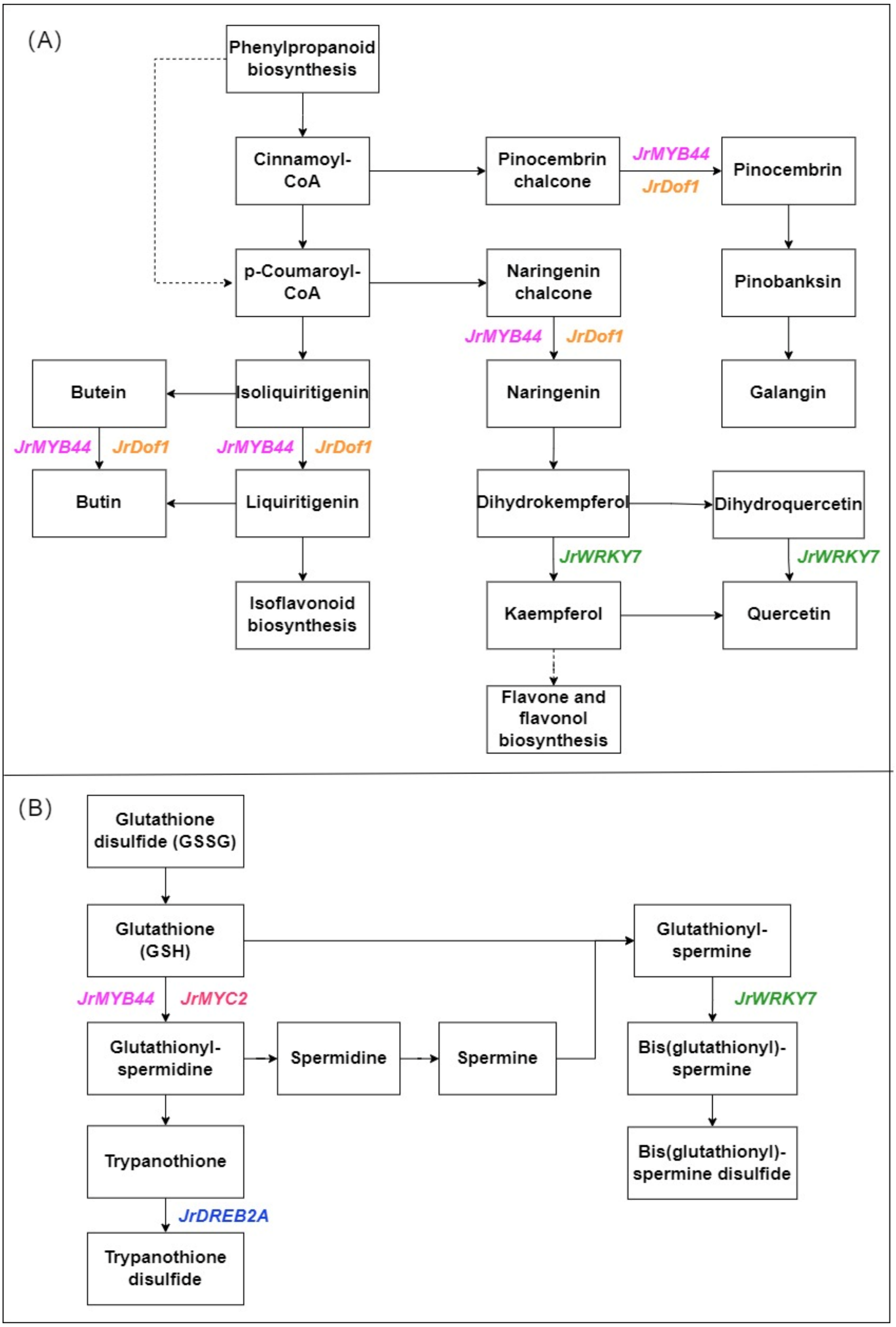

Supplementary Fig. S12 The KEGG pathway based on the transcriptomes of *JrMYB44* overexpression line and WT. A, the flavonoid biosynthesis pathway covering *JrMYB44*, *JrDof1* and *JrWRKY7*. B, the isoflavonoid biosynthesis pathway covering *JrMYB44* and *JrMYC2*, *JrWRKY7*, *JrDREB2A*.

(A)

Distribution of the top 2 Blast Hits on 1 subject sequences

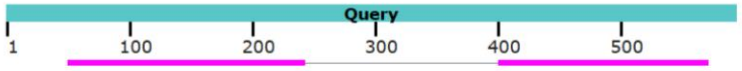

Range 1: 300 to 469 Graphics

Score	Expect	Method	Identities	Positives	Gaps
169 bits(427)	3e-50	Compositional matrix adjust.	84/179(47%)	121/179(67%)	17/179(9%)

```
Query  400  RKRGRKPANGREEPLNHVEAERQRREKLNQRFYSLRAVVPNVSKMDKASLLGDAISYINE  459
            +KRGRK   GR+ P+NHVEAERQRREKLN RFY+LR+VVPNVSKMDKASLL DA+ YINE
Sbjct  300  KKRGRKATTGRDSPVNHVEAERQRREKLNHRFYALRSVVPNVSKMDKASLLADAVVYINE  359

Query  460  LKSKLQQAESDKEEIQKKLDGMSKEGN--------NGKGCGSRAKERKSSNQDSTASSIE  511
            LK+K+       ++++ KL+   +E N        + +   S     + S+   TA++I
Sbjct  360  LKAKI-------DDLEVKLEAQPREANMSNLSGMYDSRSITSTVDHTRLSSSSFTAAAIY  412

Query  512  MEIDVKIIGWDVMIRVQCGKKDHPGARFMEALKELDLEVNHASLSVVNDLMIQQATVKM  570
            +E  VKI+G + +IR+QC   ++P AR M+AL++L+ ++ HAS+S +  LM+Q   VK+
Sbjct  413  VE--VKIVGSEALIRIQCPDVNYPHARLMDALRDLEFQIRHASISSIKGLMVQDVVVKV  469
```

Range 2: 24 to 214 Graphics

Score	Expect	Method	Identities	Positives	Gaps
142 bits(358)	6e-41	Compositional matrix adjust.	79/200(40%)	117/200(58%)	16/200(8%)

```
Query  51   TLQQRLQALIESAGENWTYAIFWQISHDFDSSTGDNTVILGWGDGYYKGEEDKEKKKNNT  110
            T+QQRLQ +I+S  E W Y+IFWQ S D      +  V+L WGDG+++G  D   K +N
Sbjct  24   TIQQRLQFIIQSRPEWWIYSIFWQTSKD-----SNGQVVLSWGDGHFRGSRDFVSKVSND  78

Query  111  NTAEQEH------RKRVIR-ELNSLISGGIGVSDESNDEEVTDTEWFFLVSMTQSFVNGV  163
               +Q        R RV +  L SL +  + V   + D +VTD+EWF+ VS+T+SF
Sbjct  79   KGDDQPRYGFGLDRTRVSKGVLQSLFAEDLDVDGVTLDGQVTDSEWFYAVSVTRSFAVRD  138

Query  164  GLPGESFLNSRVIWLSGSGALTGSGCERAGQGQIYGLKTMVCIATQNGVVELGSSEVISQ  223
            G+ G ++     IWL+G   L    C+RA + +++G++T++C+AT  GVVELGSSE I +
Sbjct  139  GVLGRAYSCGEYIWLAGDLELQFYECDRAREARLHGIQTLICVATSRGVVELGSSESIRE  198

Query  224  SSDLMHKVNNLFNFNNGGGN  243
               L+ +V +LF    G GN
Sbjct  199  DWGLVQQVKSLF----GAGN  214
```

(B)

Distribution of the top 1 Blast Hits on 1 subject sequences

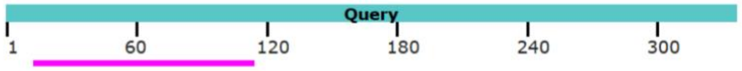

Range 1: 12 to 111 Graphics

Score	Expect	Method	Identities	Positives	Gaps
107 bits(268)	8e-32	Compositional matrix adjust.	51/101(50%)	64/101(63%)	1/101(0%)

```
Query  14   KGAWTAEEDKKLISYIHEHGEGGWRDIPQKAGLKRCGKSCRLRWANYLKPDIKRGEFSYE  73
            KG W+ EED+ L   +   G   W  I  KA   R GKSCRLRW N L P+++   F+ E
Sbjct  12   KGPWSPEEDEMLRKLVQSQGARSWSVI-SKAIAGRSGKSCRLRWCNQLSPEVEHRPFTLE  70

Query  74   EEQIIIMLHASRGNKWSVIARHLPKRTDNEIKNYWNTHLKK  114
            E+QII+  HA  GNKW+ IAR L  RTDN IKN+WN+ LK+
Sbjct  71   EDQIIVKAHAKYGNKWATIARLLNGRTDNAIKNHWNSTLKR  111
```

Supplementary Fig. S13 Amino acid sequence alignment using blastp of NCBI. A, JrMYC2 (Sbjct) and AtMYC3 (Query). B, JrMYB44 (Sbjct) and AtMYB29 (Query).
